# Better than my neighbor? Testing for overconfidence in COVID-19 preventive behaviors in Latin America

**DOI:** 10.1186/s12889-022-13311-9

**Published:** 2022-05-18

**Authors:** Cynthia Boruchowicz, Florencia Lopez Boo

**Affiliations:** 1 School of Public Policy, University of Maryland, Van Munching Hall, 7699 Mowatt Ln, College Park, MD 20740 USA; 2 Inter-American Development Bank, 1300 New York Avenue NW, Washington, DC 20577 USA

**Keywords:** overconfidence, Latin America, COVID-19, preventive behaviors

## Abstract

**Background:**

Procrastination and lack of attention may often hinder the implementation of preemptive actions necessary to mitigate the spread of COVID-19 like washing hands, covering nose and mouth with a mask, and keeping social distance. It is in such “easy” tasks that people (mistakenly) believe that they are better than others. In this paper we test for overconfidence bias in COVID-19 preventive behaviors in Latin America.

**Methods:**

Using a phone survey in nationally representative samples from 10 Latin American countries where randomly, half of the sample in each country was asked about self-reported compliance to COVID-19 guidelines, and half about preventive behavior of fellow citizens compared to them; we tested: if the proportion of individuals claiming that others comply with a certain measure “Always more frequent than me” is higher than those stating that they “Never” or “Sometimes” comply with the same measure (i.e. people believe they are better at doing something than what they actually are).

**Results:**

Over 90% of Latin-Americans claim to always wear a mask and sanitize their hands and more than 80% state to always keep social distance. We also find evidence of overconfidence in every behavior – except for keeping distance in public transportation. Moreover, the magnitude of such overconfidence is higher for behaviors such as wearing masks in public or washing hands than for those regarding keeping the 2-m distance.

**Conclusions:**

To our knowledge, this is the first study to measure overconfidence in COVID-19 preventive behaviors in Latin America. Results show that more effort is needed to encourage people to comply with the regulation when it does not only depend on them: a better organization of closed stores and public transportation are, for instance, crucial to allow social distancing. It also suggests that a reinforcement of basic measures is essential, as individuals report to be performing them more frequently than when they have to think about such behaviors compared to others.

**Supplementary Information:**

The online version contains supplementary material available at 10.1186/s12889-022-13311-9.

## Background

Procrastination and lack of attention may often hinder the implementation of preemptive actions necessary to mitigate the spread of COVID-19 like washing hands, covering nose and mouth with a mask, and keeping social distance. The use of behavioral insights gains relevance in the pandemic, as it is exactly in such “easy” tasks as the ones mentioned above that people (mistakenly) believe that they are better than others [1]. Overconfidence is particularly prevalent among young adults, and it does not go away with learning and experience [2]. In fact, research has shown that individuals who are beginners and have never performed a task (that is, the truly incompetent) are often well aware of their disability. However, with a little learning, beginners quickly believe they know a lot, if not everything, there is to know: they hold a misleading assessment of their abilities and think they are better than others [3]. Overconfidence also leads to more risk- taking behavior [4]. When we are overly confident, we form perceptions about the future (and, in general, about the probability of uncertain events occurring), in ways that may seem unreasonable. For example, previous research has identified that people tend to believe they are more skillful and safer drivers than the median driver [5]. They also expect a higher-than-average probability of having high starting wages, job satisfaction, long marriages, gifted children, and other positive life events [6, 7], but report a below-average risk of being mugged or assaulted, or of experiencing unemployment, job loss, or health problems [6, 8–13].

This anomaly can lead us to make wrong decisions, which is particularly worrisome concerning COVID-19: people might think they are better at performing preventive behaviors that they actually are. The previous has individual consequences like being more at risk of contracting and spreading the virus, as well as social ones: lower levels of (correct) compliance does not help health system to cope with an exponential demand. This is particularly important for developing countries with weaker health systems [14].

Individual data from a large-scale survey of 58 countries around the world between March and April of 2020 show that almost 90% reported to comply with government pandemic policies [15]. Stricter government restrictions increased compliance, as well as feeling personally at risk, and trust in the government doubled the impact of those restrictions on compliance – seen both in authoritarian and democratic countries [15, 16]. Nevertheless, studies show high degrees of overconfidence in the levels of performance of such compliance. During the first week of the pandemic in the United States, on average, people reported engaging in many forms of protective behavior – however, they tended to perceive their personal risk of infection as being lower than the average person in their neighborhood, state and country [16]. The same was seen in May 2020: for the three-month horizon the expected personal risk of exposure to COVID-19 was almost 18 percentage points lower than the public one, and that faded over time [17]. An online experiment performed in August 2020 showed that people with less information and understanding of COVID-19 were more overconfident and less likely to take preventable actions [18]. In Germany, robust overconfidence was found related to doing more than others to prevent the infection, having fewer negative consequences in case of infection than others, and in the probability of being hospitalized as a result of the virus – at the same time, underconfidence in the probability of getting COVID-19 was also present [19]. Finally, in Bangladesh, it was found that individuals not only think they have a higher level of awareness than what their behavior indicates, but also, think they and their family members are less prone to transmission than their neighbors [20].

In Latin America, where the massive availability of vaccines might not come as fast as in developed countries (as of September 2021, 75% of individuals in the region had yet to be fully vaccinated against COVID-19 [21]), making sure that people correctly wear masks, wash their hands and keep proper social distance at a high rate over time is key to slow the spread of the virus. However, there has been some reporting of inconsistent compliance with these measures [22]. While several structural characteristics of the region could be responsible for such inconsistency (income inequality and large populations engaged in informal work with precarious living standards, high urbanization levels with congregate settings, political instability, and cultural norms characterized by close personal relationships [23]), Latin-Americans have been found to perceive themselves as more knowledgeable that what they really are in issues like, for example, political knowledge [24]. We therefore exploit a phone survey conducted by the Inter-American Development Bank (IDB) in 10 countries to: i) study the perceptions of Latin Americans regarding the compliance with general COVID-19 guidelines, as well as specific behaviors under different circumstances; ii) test for overconfidence bias. To our knowledge, this is the first study to measure both compliance with specific preventive behaviors and overconfidence in preventive behavior in Latin America in the context of COVID-19.

## Methods

### Study design and population

The IDB conducted a phone survey through local data collection firms in 10 countries in Latin America: Uruguay, Chile, Paraguay, Peru, Ecuador, Panama, Honduras, Costa Rica, El Salvador, and Mexico. In each country 1000 individuals^1^ over 18 years old randomly chosen from a phone number database answered an 18-minutes-long questionnaire.^2^ Data collection lasted from July 29th 2020 until September 27th 2020. The survey included questions regarding technology usage, trust, behavior, and COVID-19, as well as basic sociodemographic indicators. This paper makes use of the fact that randomly in each country, half of the respondents in the survey were assigned to a questionnaire that asked about self-reported perception of compliance to COVID-19 guidelines, use of masks, hand washing and social distancing of fellow citizens compared to them in the preventive behavior module (called the *“Others”* group), and half were assigned to a questionnaire about self-reported personal behavior related to compliance to COVID-19 guidelines, use of masks, hand washing and social distancing in the preventive behavior module (called the *“Self”* group). For example:

- ***Others****: Last week, mow much MORE frequent than you did the rest of your fellow co-citizens comply with the use of masks in public? Always, Sometimes or Never?*
- ***Self****: Last week, how often did you comply with the use of masks in public? Always, Sometimes or Never?*

See Section A in the Online Resource 1 for details on data collection timeframes and survey design, and Section B in the Online Resource 1 for a description of sampling strategy.

### Variables and Measurement

**Fig. 1:**
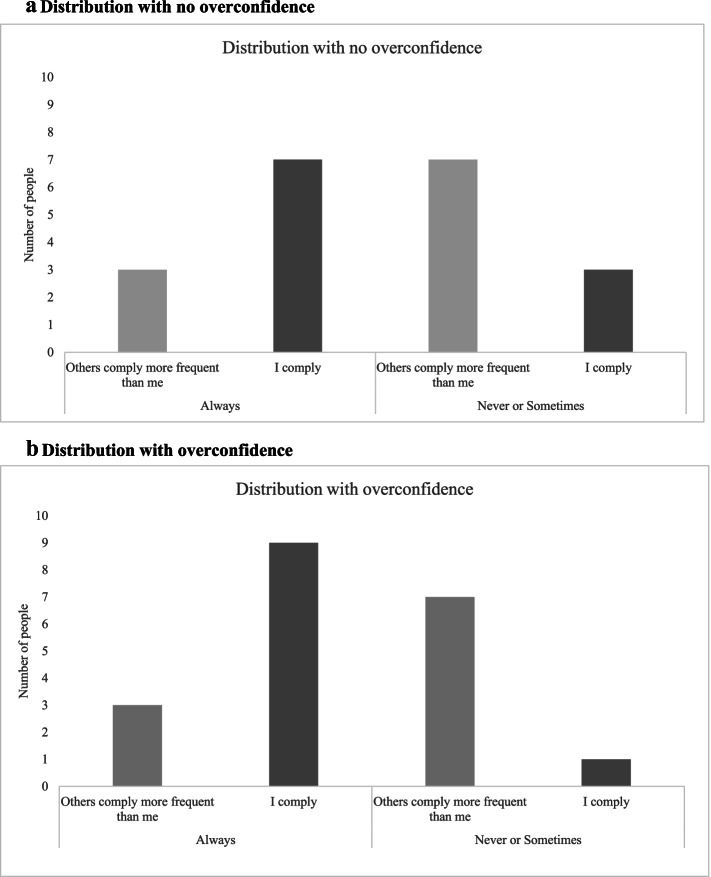
Possible distribution of compliance with preventive measures

Besides analyzing general compliance with preventive behaviors, we use the data to test for overconfidence. We do so by analyzing the proportion of individuals in the “*Others*” group that state that “Always” their co-citizens comply with the use of masks in public more frequent than them (or other preventive measure) and comparing it to the proportion of individuals in the “*Self*” group who state that they “Never” or “Sometimes” comply with the same measure ([5, 6, 8–12]). If the second figure is lower than the first, we assume there is overconfidence: when asked about themselves individuals say they do not comply with the preventive behavior less frequently than when asked about themselves in reference to others (or, in other words, when asked about themselves individuals say they comply more or better with the preventive behavior than when asked about themselves in reference to others ).^3^ Figure 1 illustrates an example of our overconfidence measure. Imagine a population of 10 individuals asked about complying with the use of masks in public, and a distribution of responses on how much more frequent than them did the other 9 co-citizens comply with the behavior where 2 say “Never”, 5 say “Sometimes” and 3 say “Always my co-citizens comply with the use of masks in public more frequent than me”. Now imagine the same 10 people getting asked about their own behavior: how frequently did they comply with the use of masks in public. If people were to respond accurately, then 3 people have to say that they either never kept the required distance, or sometimes did it: if I say that it is always the case that the rest comply more frequently than me, it is either because I do not comply (and all the rest always did) or because I do it sometimes (and all the rest always did). Figure 1.a shows what that distribution would look like. What if that number is lower than 3? Then it is the case that when people get asked about their own behavior, they say they are better at keeping distance that when they have to think about themselves compared to others. Figure 1.b shows a possible distribution. Note that in Fig. 1.a, as expected, the number of individuals that answer “Always” across the “*Self*” and “*Others*” groups add up to 10, as well as the number of individuals that answer and “Never” or “Sometimes” across the “*Self*” and “*Others*” groups. However, in Fig. 1.b, the number of individuals that answer “Always” across groups is more than 10, which is not possible as there are only 10 individuals. We are using this as a measure of overconfidence. Note that even though in the survey individuals are either assigned to the “*Self*” or “*Others*” group, we use the fact that such assignment was random to analyze this measure.

## Results

### Demographics

The relevant sample for our analysis is the 71% of respondents who left their house the week before the interview (71.8% for those who answered questions related to own behavior and 70% for the other group, a difference that is not statistically significant). In Table 1 we show the descriptive statistics for such sample – as those are the only ones for whom the compliance with preventive behavior such as keeping the 2 mt. distance or wear mask in public can be measured. On average, respondents are 41 years old, and live in a household with four members. There is a slightly lower proportion of females in the sample. In terms of education, 43% have not graduated high school, almost 37% have a high school degree and the rest have a complete university degree or more. 47% of individuals live with a child younger than 12 years old, and a resident over 60 years old lives in almost 38% of the households. Moreover, almost 80% of the individuals in the sample are smartphone users. Table 1 also includes statistics for trust. Interpersonal trust is vital: avoiding the spread of the virus depends not only on our own behavior, but also on the behavior of other’s – particularly as countries reopen and people interact more with each other outside of their COVID-19 “bubbles”. Moreover, trusting communication by governmental authorities is also important as partisanship matters for the spread of the virus [23, 25]. On this front, the Latin American countries covered by this survey present a challenging backdrop. Over 80% of respondents believe that rather than always being able to trust the majority of people, you can never be careful enough in your interaction with others. On the aggregate for our sample, 38.5% claim not to trust the government at all. Note the variables are balanced across experimental groups, which is crucial for our overconfidence analysis as it means the two groups are not different in basic characteristics (all *p*-values were two-tailed tests, and the statistical significance level set at *p* < 0.05. All the statistical analysis were performed using STATA version 14.0). Table D.1 in the Online Resource 1 uses data from Latinobarometro to show how representative our sample is in terms of key variables.

**Table 1:** Descriptive statistics (respondents who left their house the week before)

Variable	Options	Total	Behavior		*p*-value
			Self	Others	
Basic Socio Economic Characteristics					
Age	Mean	41.351	41.463	41.24	0.616
	SD	15.405	15.255	15.555	
Sex (%)	Female	0.464	0.473	0.455	0.223
Educational level (% composition)	Less than high school	0.436	0.427	0.445	0.479
	High school	0.367	0.376	0.358	0.063
	More than high school	0.194	0.195	0.194	0.222
Household characteristics	Household size: mean	4.061	4.079	4.043	0.721
	Household size: SD	2.054	2.093	2.014	
	Households with members >60 y.o. (%)	0.375	0.368	0.382	0.907
	Households with members <12 y.o. (%)	0.466	0.445	0.487	0.129
Smartphone use	Used smartphone last week (%)	0.79	0.789	0.792	0.664
Trust					
Of the following phrases, with which one do you identify more?	You can trust the majority of people	0.176	0.169	0.183	0.139
	You can never be careful enough in your interactions with others	0.795	0.803	0.786	0.134
How much do you trust the government?	A lot	0.156	0.161	0.152	0.793
	Some	0.441	0.444	0.438	0.513
	Nothing	0.385	0.378	0.391	0.374
**N**		**7511**	**3778**	**3733**	

the *p*-values show that for each covariate, we test the differences in means across groups and see they are not significant at the 5% level - there seems not to be any significant difference in baseline characteristics

### Descriptive Analysis

**Fig. 2:**
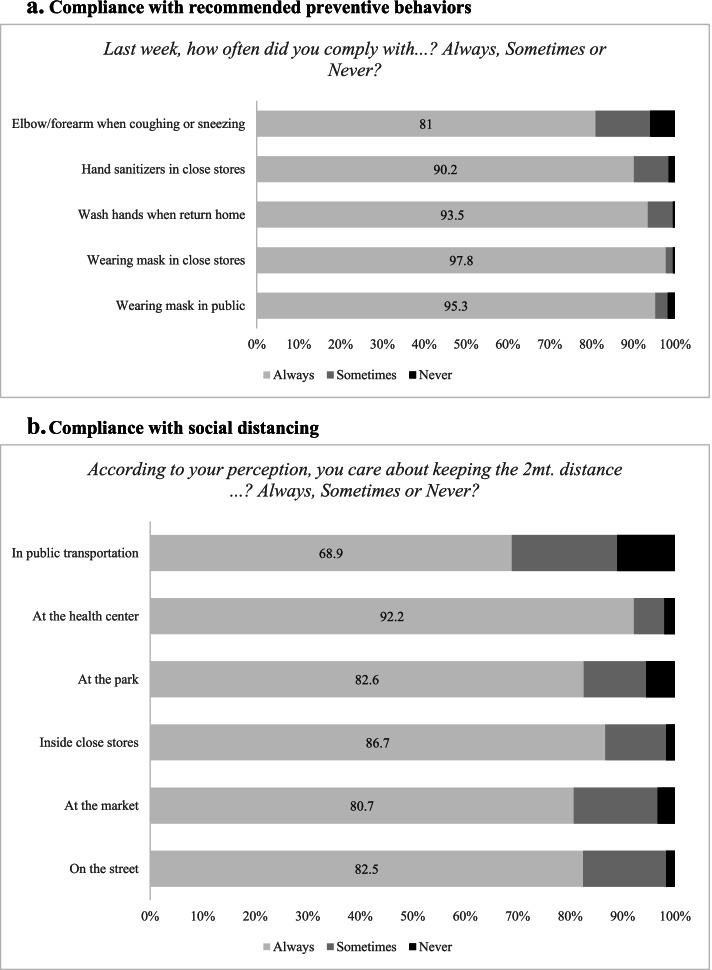
Compliance. Note: calculations based on the “*Self*” sample

Figure 2 shows compliance with use of mask, hand washing, using elbow to sneeze and keep social distance under different scenarios. Data comes from the responses of those individuals who were randomly assigned to the “*Self*” group. On average the proportion of respondents to comply with specific behaviors is high, as it was seen in international surveys [15]: over 90% claim to always use mask and sanitize their hands (see Fig. 2.a) while more than 80% state to always keep the proper social distance as shown in Fig. 2.b (except on public transportation, as expected, where the figure is almost 50%). The highest rate of compliance is for wearing masks in the market – where in the majority of the countries under analysis such action was enforced during the time of the survey – and the lowest rates of compliance are seen for keeping social distance, an action that depends on others and is not exclusively under the control of the respondents. In the Online Resource 1 a similar description can be found for compliance with general guidelines (see Section E).

**Fig. 3:**
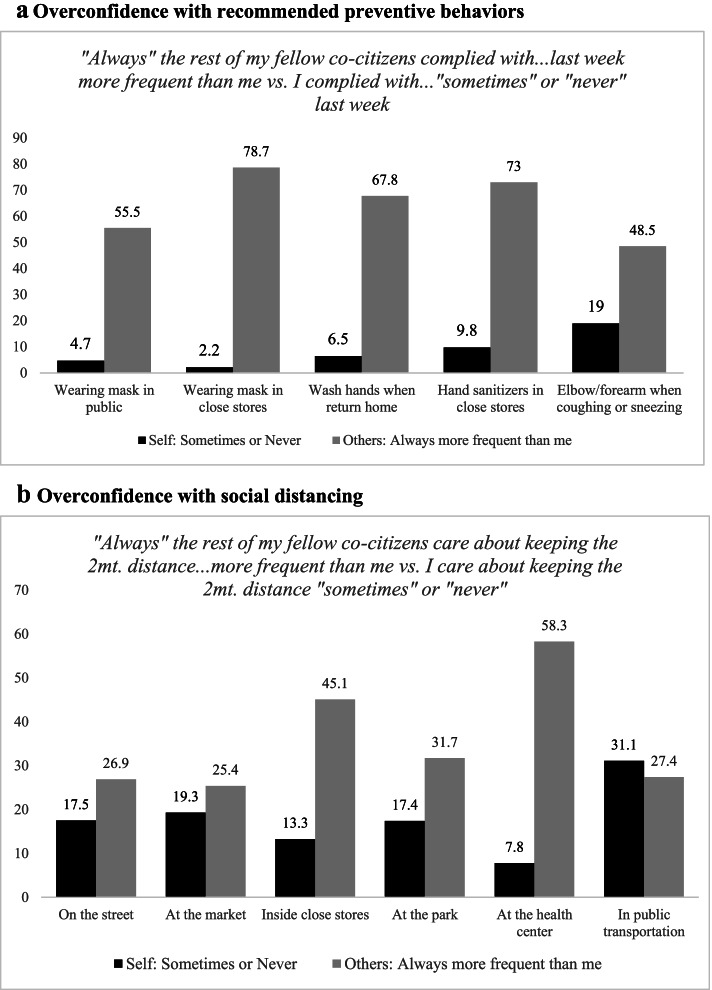
Overconfidence. Note: for overconfidence, the proportion of people stating that “Always others comply more frequent than me” has to be higher than the proportion of people who claim to comply “Never” or “Sometimes”

In line to what has been shown in other parts of the world [16–20], we see evidence of overconfidence in the compliance with preventive measures, as shown in Fig. 3 (in Fig. 3.a we see overconfidence with recommended preventive behaviors and in Fig. 3.b overconfidence with social distancing). In every case, except keeping the proper distance in public transportation, the proportion of individuals who claim that they “Never” or “Sometimes” comply with the preventive behavior or social distance in the “*Self*” group is lower than the proportion of individuals in in the “*Others*” group who claim that “Always” their co-citizens comply more frequent than them.

### Model

Taking advantage of the balance sample between the groups, we estimate overconfidence through the following econometric model:

$$\mathrm{Yi}=\beta 0+\beta 1\mathrm{Ti}+\gamma \mathbf{X}\mathrm{i}+\varepsilon \mathrm{i},$$

where Yi represents compliance with the preventive behavior: it equals 0 when individual claims to “Never” or “Sometimes” wear masks/wash hands/keep social distance or that others “Always” wear masks/wash hands/keep social distance better than them, and 1 when individual claims to “Always” wear masks/wash hands/keep social distance or that others “Never” or “Sometimes” wear masks/wash hands/keep social distance better than them. T1 is a dummy equal to 1 if subject i was randomly assigned to the “*Others*” group, and εi, is the error term. Xi is a vector of covariates including sex, age, education, household composition, level of trust in the government and level of trust in other individuals. *A negative value of β1 means overconfidence*: the proportion of respondents which state “Never” or “Sometimes” fellow citizens comply with the preventive behavior better than them is lower than the proportion of respondents to claim they “Always” comply (which is the same as stating that the proportion of respondents to claim that “Always” fellow citizens comply with the preventive behavior better than them is higher than the proportion of respondents to claim they “Never” or “Sometimes” comply, which is our measure of overconfidence). All the estimations were done with a Linear Probability Model and using an R-squared as a measure of goodness of fit. In Section F of the Online Resource 1 we follow a Logit regression and find similar results.

### Preventive behaviors (mask wearing, washing hands, sneeze on elbow)

Table 2 corroborates our visual findings. In columns (1)–(5) we can see there is evidence of overconfidence in every preventive behavior, as β1 is negative and significant. Being part of the “*Others*” group decreases the probability of compliance in between 30 to 75 p.p.: when asked about themselves individuals say they comply more or better with the preventive behavior than when asked about themselves in reference to others. It is interesting to see that demographics seem not to play in role in compliance, except for age in sneezing/coughing in the elbow (younger individuals present lower levels compared to those who are 61 or older as shown in previous studies [15]) and for education in sneezing/coughing and hand sanitization (compliance is lower for lower levels of education than for those who have more than high school completed, also in line with previous research [15]). The presence of children under 12 and seniors in the household does not seem to play a role in compliance, as well as trust (both in the government or interpersonal).

**Table 2:** Compliance with recommended preventive behaviors and social distancing

	(1)	(2)	(3)	(4)	(5)	(6)	(7)	(8)	(9)	(10)	(11)
	Compliance with preventive behaviors					Compliance with social distancing					
	Mask in public	Mask in store	Wash hand	Hand sanitizer in store	Sneez on elbow	On the street	At the market	At the store	At the park	At health center	In public transport
Others (β1)	−0.486***	−0.763***	−0.604***	−0.629***	−0.292***	−0.0870***	−0.0636***	−0.312***	−0.144***	−0.504***	0.0327*
	(0.0123)	(0.0103)	(0.0122)	(0.0119)	(0.0142)	(0.0125)	(0.0135)	(0.0134)	(0.0153)	(0.0136)	(0.0185)
18–30 years old	−0.00766	−0.000250	0.00983	−0.000173	0.0560*	−0.0223	0.000319	0.0139	0.0417	0.0556*	0.0840**
	(0.0249)	(0.0194)	(0.0242)	(0.0252)	(0.0304)	(0.0274)	(0.0286)	(0.0281)	(0.0368)	(0.0292)	(0.0390)
31–40 years old	0.00869	−0.00340	0.0476*	−0.0103	0.0750**	0.0397	0.0553**	0.0410	0.0819**	0.0910***	0.113***
	(0.0255)	(0.0212)	(0.0277)	(0.0253)	(0.0318)	(0.0266)	(0.0279)	(0.0293)	(0.0379)	(0.0323)	(0.0398)
41–50 years old	−0.00973	−0.00717	0.0258	−0.00947	0.0747**	0.0283	0.0260	0.0461*	0.0633*	0.0520*	0.0902**
	(0.0244)	(0.0196)	(0.0237)	(0.0244)	(0.0306)	(0.0265)	(0.0285)	(0.0277)	(0.0384)	(0.0294)	(0.0404)
51–60 years old	0.0102	−0.0186	0.0141	−0.0206	0.0916***	0.0597**	0.0182	0.00887	0.0425	0.00278	0.0314
	(0.0261)	(0.0214)	(0.0234)	(0.0256)	(0.0312)	(0.0269)	(0.0303)	(0.0291)	(0.0400)	(0.0299)	(0.0453)
Women	0.0163	0.00866	0.00236	0.0185	0.0248*	0.0277**	0.0255*	0.0174	0.0101	−0.00482	0.0231
	(0.0123)	(0.0103)	(0.0119)	(0.0119)	(0.0142)	(0.0125)	(0.0134)	(0.0134)	(0.0160)	(0.0146)	(0.0179)
Less than High School	−0.0245	−0.00215	−0.0501***	−0.0427***	−0.0877***	−0.0866***	−0.0974***	−0.0932***	−0.0527**	−0.0966***	−0.109***
	(0.0155)	(0.0130)	(0.0155)	(0.0151)	(0.0179)	(0.0157)	(0.0168)	(0.0169)	(0.0205)	(0.0188)	(0.0222)
High School	0.00326	−0.00234	−0.0241**	−0.0468***	−0.0477***	−0.0375***	−0.0500***	−0.0472***	−0.0500***	−0.0419***	−0.0766***
	(0.0129)	(0.00932)	(0.0120)	(0.0124)	(0.0146)	(0.0127)	(0.0140)	(0.0139)	(0.0171)	(0.0154)	(0.0184)
Child<12 present	0.0000770	−0.00953	−0.0240*	0.00127	−0.0111	−0.00230	−0.00480	−0.00429	0.00216	−0.00585	−0.00896
	(0.0130)	(0.00978)	(0.0130)	(0.0121)	(0.0152)	(0.0133)	(0.0142)	(0.0145)	(0.0167)	(0.0153)	(0.0200)
Senior present	−0.00838	−0.00809	−0.00132	−0.0159	−0.00209	−0.0115	0.00364	−0.00346	−0.00616	0.00534	0.0150
	(0.0150)	(0.0115)	(0.0155)	(0.0143)	(0.0174)	(0.0155)	(0.0160)	(0.0164)	(0.0185)	(0.0177)	(0.0205)
Trust Gov.	0.0141	−0.00387	0.0145	−0.0142	−0.0106	−0.0237	−0.00486	−0.0281*	−0.0164	−0.0482***	−0.0480**
	(0.0144)	(0.0119)	(0.0134)	(0.0137)	(0.0159)	(0.0145)	(0.0151)	(0.0152)	(0.0181)	(0.0166)	(0.0202)
Trust others	−0.0170	0.00375	−0.0198	−0.0123	−0.0248	−0.0333*	0.0177	0.00692	−0.0171	0.00268	0.00119
	(0.0156)	(0.0119)	(0.0157)	(0.0156)	(0.0182)	(0.0173)	(0.0177)	(0.0178)	(0.0220)	(0.0182)	(0.0261)
Constant	0.907***	1.000***	0.984***	0.981***	0.834***	0.877***	0.851***	0.945***	0.853***	0.987***	0.752***
	(0.0370)	(0.0332)	(0.0380)	(0.0396)	(0.0430)	(0.0367)	(0.0370)	(0.0388)	(0.0468)	(0.0425)	(0.0533)
Observations	7337	7191	7084	7070	7015	7309	6377	7084	4654	5276	4725
FE by country	YES	YES	YES	YES	YES	YES	YES	YES	YES	YES	YES
R-squared	0.291	0.617	0.419	0.420	0.123	0.038	0.027	0.142	0.041	0.301	0.034

Results of Linear Probability Model. Robust standard errors in parenthesis. Population weights used.* *p* < 0.10 ** *p* < 0.05 *** *p* < 0.01. Marginal effects displayed (a coefficient of 0.1 implies it is 10 p.p. more likely to comply with the preventive behavior the base category). Base categories: belonging to the “Others” group, 61 years old or more, more than HS (high school); men; no children under 12 at home; no seniors at home; no trust. Includes country FE

### Social Distancing

Regarding keeping distance, Columns (6)–(11) in Table 2 show that there is evidence of overconfidence on the street, at the market, at the store, at the park and at the health center. The lower levels are found at the street and the market. The previous is expected given that there is more space to social distance at those places, and it was also very publicized by health officials [26]. Moreover, it can be seen that there is no evidence of overconfidence in keeping distance at public transport, which is expected as well as this survey was conducted between July and September 2020 when there were still restrictions to mobilization (note that the sample size for this estimation is lower than for the rest of the behaviors analyzed). Compliance in this case also decreases for younger cohorts and those with lower levels of education. The presence of children under 12 and seniors in the household also does not seem to play a role in compliance, as well as interpersonal trust. Compliance with the proper distance at the health center and in public transport is lower for those who trust the government.

Overall, it is interesting to note that we do not see effects of household composition or trust on our compliance measures contrary to what was expected. Previous research has shown that trust does not play a role by itself on compliance, but rather by making a difference in the stringency level of compliance [15]. In this case, as data was collected between July and September 2020, countries where still showing high levels of strictness in the COVID-19 guidelines and such luck of variation within the region might be explaining the results. In terms of household composition, in Table 3 we show the results of the estimations but with the interaction between the group dummy and the presence of a child under 12 and of a senior at home. While household composition seems not to play a direct role in compliance, it does through its effect on overconfidence. As expected, having a child under 12 or a senior at home increases the effect of the “*Others*” dummy: when asked about themselves individuals say they comply more or better with the preventive behavior than when asked about themselves in reference to others and that effect is larger for those with at-risk individuals at home.

**Table 3:** Interaction Effects

	(1)	(2)	(3)	(4)	(5)	(6)	(7)	(8)	(9)	(10)	(11)
	Compliance with preventive behaviors					Compliance with social distancing					
	Mask in public	Mask in store	Wash hand	Hand sanitizer in store	Sneez on elbow	On the street	At the market	At the store	At the park	At health center	In public transport
Others (β1)	−0.424***	−0.743***	−0.558***	−0.608***	−0.278***	−0.0420**	−0.0254	−0.249***	−0.109***	−0.476***	0.0819***
	(0.0193)	(0.0152)	(0.0184)	(0.0186)	(0.0214)	(0.0189)	(0.0215)	(0.0214)	(0.0247)	(0.0215)	(0.0280)
Child<12 present	0.0395***	0.00124	0.00647	0.0181	0.00687	0.0268	0.0163	0.0361**	0.0318	−0.00497	0.0236
	(0.0115)	(0.00798)	(0.0125)	(0.0134)	(0.0180)	(0.0165)	(0.0181)	(0.0157)	(0.0203)	(0.0139)	(0.0286)
Others x Child<12 present	−0.0801***	−0.0219	−0.0638***	−0.0346	−0.0361	−0.0592**	−0.0425	−0.0815***	−0.0549*	−0.00291	−0.0607
	(0.0249)	(0.0208)	(0.0244)	(0.0242)	(0.0286)	(0.0255)	(0.0271)	(0.0271)	(0.0313)	(0.0282)	(0.0369)
Senior present	0.0259**	0.00516	0.0202	−0.00910	−0.00672	0.0132	0.0297	0.0318*	0.00846	0.0449***	0.0465*
	(0.0131)	(0.00899)	(0.0138)	(0.0146)	(0.0192)	(0.0179)	(0.0191)	(0.0168)	(0.0230)	(0.0151)	(0.0280)
Others x Senior present	−0.0677***	−0.0262	−0.0435*	−0.0134	0.00932	−0.0487*	−0.0513*	−0.0701**	−0.0271	−0.0734**	−0.0577
	(0.0254)	(0.0205)	(0.0255)	(0.0250)	(0.0300)	(0.0265)	(0.0283)	(0.0280)	(0.0329)	(0.0286)	(0.0366)
Constant	0.875***	0.990***	0.961***	0.971***	0.829***	0.854***	0.831***	0.913***	0.834***	0.970***	0.723***
	(0.0373)	(0.0337)	(0.0386)	(0.0396)	(0.0431)	(0.0374)	(0.0385)	(0.0395)	(0.0485)	(0.0420)	(0.0552)
Observations	7337	7191	7084	7070	7015	7309	6377	7084	4654	5276	4725
FE by country	YES	YES	YES	YES	YES	YES	YES	YES	YES	YES	YES
Controls	YES	YES	YES	YES	YES	YES	YES	YES	YES	YES	YES
R-squared	0.294	0.617	0.420	0.420	0.123	0.040	0.028	0.145	0.042	0.303	0.035

Results of Linear Probability Model. Robust standard errors in parenthesis. Population weights used.* *p* < 0.10 ** *p* < 0.05 *** *p* < 0.01. Marginal effects displayed (a coefficient of 0.1 implies it is 10 p.p. more likely to comply with the preventive behavior the base category). Base categories: belonging to the “Others” group, 61 years old or more, more than HS (high school); men; no children under 12 at home; no seniors at home; no trust. Includes country FE

In the Online Resource 1 (Section F) we perform two robustness checks that solidify our results. First, a concern is that individuals might not have understood the question referring to “how much more frequent than you did the rest of your fellow citizens comply with the use of masks, hand wash or social distance”, and they simply replied to how much they believe others comply with the preventive measure. We therefore run such a model: we compare the proportion of individuals who state that they “always” comply with those that state that others “always comply better than me” (assuming people were answering such questions as “others always comply”). We consistently find that the first value is larger than the second: when we ask individuals about their own behavior, they say they comply at a higher rate than when asked about the behavior of others. Given that that two samples are balanced, that could not happen, and it is evidence for overconfidence. Secondly, and to address a possible social desirability bias, we change the outcome to different governmental surveillance methods to guarantee that those COVID-19 positive in fact quarantine. According to Wise et al. [16], feeling personally at risk is the most important predictor for engaging in preventive behaviors in the context of COVID-19. Then, the rate at which individuals support different surveillance methods should be higher if the one under quarantine is a random person than if it is oneself: the risk of no surveillance for the first means an increased probability of exposure for the respondent, while the second is a strict control over one’s own movements. Nevertheless, there are not significant differences in support for different surveillance methods between the groups.

## Discussion

“People realize the risk of getting COVID-19 from suboptimal behaviors such as not washing hands or not adhering to social distancing but are likely to believe that they are *less* likely than other people or their peers to get COVID-19, even if their peers adhere to preventive practices” [27 , p. 347]. In Latin America, where the massive availability of vaccines is not coming as fast as in developed countries, adherence to preventive practices at a high rate over time is key to contain the spread of the virus. In this paper, we show that self-reported compliance with local guidelines is high in 10 Latin-American countries: Over 90% claim to always use mask and sanitize their hands while more than 80% state to always keep the proper social distance. As expected, the highest rate of compliance is for wearing masks in markets – where in the majority of the countries under analysis such action was enforced during the survey timeframe – and the lowest rates of compliance are seen for keeping social distance, an action that also depends on others and is not exclusively under the control of the respondent. These findings go in line with Moloney [28], which found that 7% of Latin Americans never wear a mask when leaving their house, and are similar to what was seen in the rest of the world [15].

This paper is also, to our knowledge, the first study to measure overconfidence in preventive behaviors in Latin America in the context of COVID-19. In our sample, there is evidence of overconfidence in every behavior – except for keeping distance in public transportation. Moreover, the magnitude of such overconfidence is higher for behaviors such as wearing masks in public or washing hands than for those regarding keeping the 2-m distance. The previous is expected given that keeping distance is a situation that many times is out of the individual’s control and depends on the action of others as well. Particularly in the case of public transportation, it is important to highlight that the survey was conducted between July and September 2020, when there were still restrictions to mobilization. Our results are in line to those in United States [16–18], Germany [19], and Bangladesh [20]. We also found that having a child under 12 or a senior at home increases the effect of overconfidence in compliance with preventive measures.

We acknowledge limitations in our study. First, it is possible that our results reflect social desirability bias – people respond what they think they should respond. Even though we do not see differences in support for surveillance measures between the groups when the one under quarantine is a stranger versus the respondent himself, it is not possible to completely eliminate this possibility. Second, it is possible that people think they are complying with the guidelines but are not doing it properly – it is still usual, for example, to see people wearing their masks under their nose. Third, people might have not understood well the question that referring to how much more frequent than you did the rest of your fellow citizens comply with the use of masks, hand wash or social distance*.* Even though we tested the model assuming that individuals responded about other’s behaviors (and not other behaviors compared to themselves) and found similar results, we cannot completely disregard this possibility and the “noise” it could have created on our overconfidence measure. Moreover, it is important to highlight that even though the urgency of the COVID-19 pandemic meant that several studies relied on self-reported behaviors to give policy recommendations, we are not testing observed behaviors (note, however that previous research projects have found a good correlation between self-reported and actual behavior [29, 30]). Finally, data from this study comes from a survey performed during July to September 2020, when most restrictions in the countries where still in place.

There are different policy lessons from our study which are important for the context of Latin America. Our results suggest not only that public health campaigns have seemed to work in the region and compliance is high, but also that more effort from governments is needed to encourage people to comply with the regulation when it does not only depend on them: a better organization of closed stores and public transportation are, for instance, crucial to allow social distancing. These renewed public efforts will be key in the future as countries start giving up the last COVID-19 restrictions, but some still lag in vaccination rates. It also suggests that a reinforcement of basic measures like mask wearing, and hand sanitization is essential. Individuals report to be wearing masks, washing their hands or coughing at their elbow more frequently than what they actually are (as shown in our results when individuals have to think about such behaviors compared to others). This may have not only individual consequences, but also for the society as a whole. For instance, lower levels of (correct) compliance does not help health system to cope with an exponential demand. It also impacts school attendance for unvaccinated children, return to work of working mothers due to childcare closures and can even have consequences in the normal functioning of the production system of the economy as those who have been infected have to quarantine. Therefore, as governments in the region re-launch their campaigns to balance openings, vaccination efforts and containing the spread of the virus and its new variants, it is important that they make the effort to keep reinforcing how to correctly perform basic measures in order to counterbalance overconfidence which most times lead to the assumption of excessive risks.

## Conclusions

We found in this paper that self-reported compliance with preventive behaviors is high in Latin America: over 90% of individuals claim to always wear a mask and sanitize their hands and more than 80% to always keep social distance. Moreover, and in line with the results from other regions, we find evidence of overconfidence in the compliance with every preventive behavior except for keeping distance in public transportation. The magnitude of overconfidence is higher for behaviors such as wearing masks in public or washing hands than for those regarding keeping the 2-m distance. The previous is expected given that keeping distance is a situation that many times is out of the individual’s control and depends on the action of others as well. These findings imply not only that public health campaigns have seemed to work in the region, but also that more effort from governments is needed to encourage people to comply with the regulation when it does not only depend on them: a better organization of closed stores and public transportation are, for instance, crucial to allow social distancing. It also suggests that a reinforcement of basic measures like mask wearing, and hand sanitization is essential, as individuals report to be wearing masks, washing their hands or coughing at their elbow more frequently than what they actually are (seen when they have to think about such behaviors compared to others). To our knowledge, this is the first study to measure both compliance with specific preventive behaviors and overconfidence in preventive behavior in Latin America in the context of COVID-19. Data from this study comes from a survey performed during July to September 2020, when most restrictions where still in place in Latin American countries. Therefore, performing the same analysis with more updated data could shed light on future efforts from policymakers as the region and the world enter a new phase of the pandemic.

## Supplementary Information

**Additional file 1.** Online Resource 1. Survey Details, Sample Description, Preventive Behavior Distribution between “Others” and “Self” groups, Sample Robustness, Results of compliance with local guidelines, Additional Estimations, Survey instrument (in Spanish).

## Data Availability

Public access to the data that supports the findings of this study is closed. The data is property of the Inter-American Development Bank. Restrictions apply to the availability of this data, and so it is not currently publicly available. The custom codes generated during the current study are available from the corresponding author on reasonable request.

## Footnotes

1 In each country, the sample was of 1000 individuals to make the survey representative at the national level. Only in Mexico the sample was of 1200 individuals to make it representative at the state level.

2 The data collection happened via phone due to the COVID-19 pandemic, and verbal informed consent was obtained prior to the interview (enumerators explained that within the framework of an international study on the health emergency caused by the coronavirus and the tools to reduce contagion, they were asking these questions –that their response was voluntary and strictly confidential, and specifically asked “Do you agree to participate?”). Three data collection firms carried out the survey. Phone numbers were randomly selected through Random Digital Dialing from a sampling frame constructed either on enables prefixes from mobile phone companies or a database of numbers previously compiled in market research. No identifiable information like name, address or national ID was collected, so data in this study is anonymous.

3 Table C.1 in Section C of the Online Resource 1 shows different possible distributions of people responding to how much they complied to a preventive behavior, and what their responses should be if they were asked about how much more frequent than them did the other co-citizens comply with the behavior. It shows that the only way that someone can respond “Always” their co-citizens complied more frequent than them is if, when asked about themselves, that person responds that they either “Never” or “Sometimes” complied.
