# Supplementary material for: Better than my neighbor? Testing for overconfidence in COVID-19 preventive behaviors in Latin America
Source: BMC Public Health. 2022 May 18;22:1009. doi: 10.1186/s12889-022-13311-9 (PMC9116709; doi:10.1186/s12889-022-13311-9)
Supplement: Supplementary file 1 — Additional file 1. Online Resource 1. Survey Details, Sample Description, Preventive Behavior Distribution between “Others” and “Self” groups, Sample Robustness, Results of compliance with local guidelines, Additional Estimations, Survey instrument (in Spanish). [file 12889_2022_13311_MOESM1_ESM.docx]

**Supplementary Information (SI): Online Resource 1**

**Contents**

1. **Survey Details**
2. **Sample**
3. **Preventive Behavior Distribution between “*Others*” and “*Self*” groups**
4. **Sample Robustness**
5. **Compliance with local guidelines**
6. **Additional Estimations**
7. **Instrument (Spanish)**

**A Survey Details**

**A.1 Survey Timeframe**

The data collection process overall started on July 29th 2020 and finished on September 27^th^ 2020. See Table A.1 shows timeframes per country.

**Table A.1. Data collection process timeframe, by country (2020)**

| Country | Started | Finished | COVID-19 cases* | | COVID-19 deaths* | |
| --- | --- | --- | --- | --- | --- | --- |
|  |  |  | Started | Finished | Started | Finished |
| Chile | July 29th | August 20th | 1,868.9 | 2,069.0 | 49.6 | 56.1 |
| Uruguay | July 29th | August 22^nd^** | 35.2 | 44.1 | 1.0 | 1.2 |
| Paraguay | July 30th | August 18th | 69.1 | 143.9 | 0.7 | 2.1 |
| Peru | July 30th | August 15^th^** | 1,232.5 | 1,648.5 | 57.9 | 80.8 |
| Ecuador | August 4th | August 28th | 550.4 | 689.5 | 33.2 | 37.2 |
| El Salvador | August 6th | August 23rd | 289.2 | 380.8 | 7.9 | 10.3 |
| Honduras | August 7^th^** | September 5^th**^ | 469.5 | 665.0 | 14.8 | 20.8 |
| Costa Rica | August 8^th^** | September 26^th^** | 461.3 | 1,427.4 | 4.7 | 16.4 |
| Panama | August 9th | September 27^th^** | 1,754.2 | 2,603.5 | 38.6 | 55.1 |
| Mexico | August 31st | September 8th | 467.0 | 499.7 | 50.3 | 53.1 |

* Cases and deaths per 100,000 inhabitants

** August 10, 2020, August 24, 2020

Source: Inter-American Development Bank, Social Protection and Health Division (SPH) calculations based on European CDC data.

**A.2 Survey Design**

The survey included eight modules on questions regarding technology usage, trust, behavior, and COVID-19, as well as basic socio demographic indicators. The order of the modules is: Basic Individual Characteristics (Module 1), Basic Household Characteristics (Module 2), General Technology Usage (Module 3), Trust and COVID-19 (Module 4), Administrative Procedures (Module 5), Technology and COVID-19 (Module 6 – hypothetical app), Data Privacy (Module 7), Behavior (Module 8). Table A.2 shows the structure of the survey.

**Table A.2 Structure of the Survey**

| Module 1: Basic Individual Characteristics | Age  Sex  Education |
| --- | --- |
| Module 2: Basic Household Characteristics | Household composition |
| Module 3: General Technology Usage | Smartphone usage  Smartphone activities |
| Module 4: Trust and COVID-19 | Interpersonal trust  Trust in government  Trust information COVID-19  Support quarantine strategies |
| Module 5: Administrative Procedures | Type of administrative procedures before and after COVID-19  Restrictions to in-person services |
| Module 6: Technology and COVID-19 | Acceptance of contact tracing apps  Reasons to accept/not accept  Acceptance w/ conditionalities |
| Module 7: Data Privacy | Knowledge about personal data regulation  Control over personal data |
| Module 8: Behavior | Preventive behavior observance |

There were two versions of Module 8, that were randomly assigned among the sample. One set of questions in the module were:

- **OTHERS:** Last week, how much MORE frequent than you did the rest of your fellow co-citizens comply with the following recommendations? Always, sometimes, or never?
- **SELF:** Last week, how often did you comply with the following recommendations? Always, sometimes, or never?

| Behavior | 1  Always | 2  Sometimes | 3  Never | 98  Did not leave the house | 99  NA |
| --- | --- | --- | --- | --- | --- |
| Wearing mask in public |  |  |  |  |  |
| Wearing mask in close stores |  |  |  |  |  |
| Hand sanitizers in close stores |  |  |  |  |  |
| Wash hands when return home |  |  |  |  |  |
| Elbow/forearm when coughing or sneezing |  |  |  |  |  |

The survey then asks

- **OTHERS:** According your perception, how MORE frequent than you did the rest of your fellow co-citizens care about keeping the 2mt. distance in…? Always, sometimes or never?
- **SELF:** According to your perception, do you care about keeping the 2mt. distance in…? Always, sometimes, or never?

| Behavior | 1  Always | 2  Sometimes | 3  Never | 98  Did not leave the house | 99  NA |
| --- | --- | --- | --- | --- | --- |
| In public transportation |  |  |  |  |  |
| At the health center |  |  |  |  |  |
| At the park |  |  |  |  |  |
| Inside close stores |  |  |  |  |  |
| At the market |  |  |  |  |  |
| On the street |  |  |  |  |  |

For the complete instrument, please see section G (in Spanish, the original language)

**B Sample**

**B.1 Recruitment**

Three data collection firms were hired to carry out the survey. One firm oversaw Mexico, one Chile, Uruguay, Paraguay, and Peru and another one Ecuador, El Salvador, Honduras, Costa Rica and Panama. In each country, the sample was of 1,000 individuals to make the survey representative at the national level. Only in Mexico the sample was of 1,200 individuals to make it representative at the state level.

In Chile, a sample frame of telephone numbers of each company present in the country was generated, according to their market proportion based on statistics from the Telecommunications Undersecretariat, and then proceeded with a Random Digit Dialing system, with fixed numbers and cell phones. Random numbers were generated based on the block of first fixed digits that each company must assign to the telephone numbers of its users.15,859 attempts were made for a total number of 1,004 complete surveys. In Paraguay, the prefixes enabled for each cell phone company, which are 4 (Tigo, Personal, Claro and Vox) were used as a reference. Each company has an unequal number of prefixes, as well as market proportion differentials. The data of the market proportion of each company was taken to generate a stratified sample that considers that weight over the total (within each sample stratum – company- the sample was distributed equally according to available prefixes. 17,789 attempts were made for a total number of 1,021 complete surveys. In Peru, a sampling frame was built from the numbering series of the mobile services of the Ministry of Transport and Communications (MTC). In the country there are four providers of cell phone lines: Movistar, Claro, Entel and Bitel. The series of MTC numbers correspond to all providers and contain the same number of digits as cell phone numbers (9 digits). The first five have a header function and the last four can be numbered from "0000" to "10000". In order to have a valid sampling frame, the randomly selected numbers go through an IVR (Interactive Voice Response). The sample frame is representative of the mobile lines market and includes both old and new users, to the extent that numbers of all the headend by operator are included. 98,610 attempts were made for a total number of 1,009 complete surveys. In Uruguay, the data collection company has a sample frame of telephone numbers of the three mobile telephone companies that operate in the country, considering the prefixes used by each one. Subsequently, a Random Digit Dialing system was used to verify the existence or not of the number, and those numbers that correspond to an existing telephone number were kept. 22,000 attempts were made for a total number of 1,012 complete surveys. In Honduras, Costa Rica and El Salvador the data collection company already had a database of numbers previously compiled for market research. The average response rate to achieve a sample of 1,000 individuals per country was 8.2, 4.5 and 28.4, respectively (in El Salvador, the sample was of 997). In Panama the data collection company counted with a digital phone book and numbers were randomly selected through Random Digital Dialing with an average response rate of 14.2 to achieve a sample of 1,000 individuals. In Ecuador, with data from local telephone directories and Synergie's own databases collected from studies from previous years, 1,000 individuals were surveyed (response rate of approximately 60 percent).

Finally, in Mexico the data collection firm first prepared the sampling frame using the National Numbering Plan (PNN), whose administration and use of national numbering is attributed to the Federal Telecommunications Institute (IFT). In this Plan there are 617,567,770 possible total numbers that the different fixed or mobile telephone service providers in Mexico can distribute among their users, whether or not they have yet been assigned for use to a particular user. In this plan there are two main components: the national long-distance code (lada) and the serial number, which corresponds to the numbers with which a specific number starts. Among these components, six of the ten digits that make up the total number of digits required to dial a telephone number in the country are identified. For each of the combined components of lada and series, four random numbers were generated, resulting in 504,896 unique numbers. For these, an automatic dialing program (Blaster) was used to identify 101,902 telephone numbers, which were randomly divided into 12 samples for a total sample of 1,214 individuals using the Bassols-Batalla regionalization to meet the sample stratification criterion by geographic division.

**C Preventive Behavior Distribution between “*Others*” and “*Self*” groups**

Table C.1, Panel A shows different possible distributions of people responding to how much did they comply with the preventive behavior (group “*Self*”). Panel B shows what their response should be if they were asked about how much more frequent than them did the other 9 co-citizens comply with the behavior (group “*Others*”). It shows that there are only two scenarios in which someone can answer “always more frequent than me”: if the person never complies and all the other 9 always do, or if the person sometimes complies and all the other 9 always do. As such, a higher proportion of individuals claiming “always more frequent than me” than those stating they “never” or “sometimes” comply with the preventive measure means that individuals say they are better at doing something when asked about own behavior rather than when they have to frame such action compared to others.

**Table C.1. Possible preventive behavior distributions**

Panel A. *Last week, how often did you comply with the use of masks in public? Always, Sometimes or Never?*

|  | Individual | | | | | | | | | |
| --- | --- | --- | --- | --- | --- | --- | --- | --- | --- | --- |
|  | 1 | 2 | 3 | 4 | 5 | 6 | 7 | 8 | 9 | 10 |
| Situation A | Always | Never | Never | Never | Never | Never | Never | Never | Never | Never |
| Situation B | Always | Always | Always | Always | Always | Always | Always | Always | Always | Always |
| Situation C | Always | Always | Always | Always | Sometimes | Sometimes | Never | Never | Never | Never |
| Situation D | Never | Never | Never | Never | Never | Never | Never | Never | Never | Never |
| Situation E | Never | Always | Always | Always | Always | Always | Always | Always | Always | Always |
| Situation F | Never | Always | Always | Always | Sometimes | Sometimes | Never | Never | Never | Never |
| Situation G | Sometimes | Never | Never | Never | Never | Never | Never | Never | Never | Never |
| Situation H | Sometimes | Always | Always | Always | Always | Always | Always | Always | Always | Always |
| Situation I | Sometimes | Always | Always | Always | Sometimes | Sometimes | Never | Never | Never | Never |
| Panel B. Responses to: *Last week, mow much MORE frequent than you did the rest of your fellow co-citizen comply with the use of masks in public? Always, Sometimes or Never?* Based on Panel A | | | | | | | | | | |
|  | Individual | | | | | | | | | |
|  | 1 | 2 | 3 | 4 | 5 | 6 | 7 | 8 | 9 | 10 |
| Situation A | Never | Sometimes | Sometimes | Sometimes | Sometimes | Sometimes | Sometimes | Sometimes | Sometimes | Sometimes |
| Situation B | Never | Never | Never | Never | Never | Never | Never | Never | Never | Never |
| Situation C | Never | Never | Never | Never | Sometimes | Sometimes | Sometimes | Sometimes | Sometimes | Sometimes |
| Situation D | Never | Never | Never | Never | Never | Never | Never | Never | Never | Never |
| Situation E | Always | Sometimes | Sometimes | Sometimes | Sometimes | Sometimes | Sometimes | Sometimes | Sometimes | Sometimes |
| Situation F | Sometimes | Never | Never | Never | Sometimes | Sometimes | Sometimes | Sometimes | Sometimes | Sometimes |
| Situation G | Never | Sometimes | Sometimes | Sometimes | Sometimes | Sometimes | Sometimes | Sometimes | Sometimes | Sometimes |
| Situation H | Always | Sometimes | Sometimes | Sometimes | Sometimes | Sometimes | Sometimes | Sometimes | Sometimes | Sometimes |
| Situation I | Sometimes | Never | Never | Never | Sometimes | Sometimes | Sometimes | Sometimes | Sometimes | Sometimes |

Note: Panel A shows different possible distributions of people responding to how much did they comply with the preventive behavior. Panel B shows what their response should be if they were asked about how much more frequent than them did the other 9 co-citizens comply with the behavior.

**D Sample Robustness**

To be sure of the validity of our sample, we present in Table D.1 the results of the Latinobarometro 2018 wave. Note that while in our sample a higher proportion of individuals are smartphone users (73 percent versus 45 percent), both the socioeconomic composition as well as variables such as trust, social network usage and privacy concern are similar between the Latinobarometro sample and the sample we use for our analysis.

**Table D.1 Descriptive statistics, Latinobarometro (2018)**

| Variable | Option | Mean | SD |
| --- | --- | --- | --- |
| Age |  | 40.81 | 2.776 |
| Sex | Female | 0.520 | 0.0156 |
| Education level (% composition) | Primary | 0.396 | 0.167 |
|  | Secondary | 0.393 | 0.103 |
|  | Tertiary | 0.210 | 0.0950 |
| Usage of Social Media (%that mentions it) | Facebook | 0.600 | 0.100 |
|  | Twitter | 0.120 | 0.0507 |
|  | Whatsapp | 0.640 | 0.127 |
|  | No Social Networks | 0.285 | 0.108 |
| Trust in Government (%) | A lot | 0.0580 | 0.0318 |
|  | Some | 0.164 | 0.0762 |
|  | Little | 0.319 | 0.0708 |
|  | Nothing | 0.434 | 0.123 |
| Trust others (%) |  | 0.141 | 0.0447 |
| "The use of private information on the Internet for commercial purposes represents a violation of a basic human right" (%) | Agrees a lot | 0.150 | 0.0646 |
|  | Agrees some | 0.463 | 0.0795 |
|  | Agrees a little | 0.232 | 0.0530 |
|  | Does not agree | 0.0446 | 0.0247 |
| Smartphone user (%) |  | 0.452 | 0.115 |

Source: Latinobarometro (2018)

**E Compliance with local guidelines**

Figure E.1 shows the responses when individuals were asked about compliance with local guidelines to deal with the pandemic for our relevant sample by country. Overall at the extremes, almost 48 percent of respondents claim to comply with the regulations 100 percent of the time and less than 1 percent state they never comply with the rules. On average, 89 percent of respondents report to comply with the regulations more than half of the time (always, most of the times); 5 percent do so half of the time and 6 percent do so less than half of the time (sometimes, never). Figure E.1 also shows responses for the sample that was asked about their behavior relative to others. On average, 49 percent of respondents claim that less than half of the time other citizens comply with guidelines better than them; 28 percent state that half of the time others comply with the guidelines better than them, and 24 percent state that more than half of the times others comply with the guidelines better than them.

**Figure E.1. Compliance with local authorities’ recommendations**

Note: For those asked about self-behavior, the options were complying “always”, “most of the times”, “half of the times”, “sometimes” or “never”. For those in the other group, the options were others comply “always better than me”, “most of the times better than me”, “half of the times better than me”, “sometimes better than me” or “never better than me”.

**F Additional estimations**

**F.1 Results from Logit Regressions**

|  | (1) | (2) | (3) | (4) | (5) | (6) | (7) | (8) | (9) | (10) | (11) |
| --- | --- | --- | --- | --- | --- | --- | --- | --- | --- | --- | --- |
|  | Compliance with preventive behaviors | | | | | Compliance with distance | | | | | |
|  | Mask in public | Mask in store | Wash hand | Hand sanitizer in store | Sneeze on elbow | On the street | At the market | At the store | At the park | At health center | In public transport |
|  |  |  |  |  |  |  |  |  |  |  |  |
| Other (β1) | 0.0554*** | 0.00511*** | 0.0302*** | 0.0386*** | 0.242*** | 0.591*** | 0.685*** | 0.184*** | 0.445*** | 0.0547*** | 1.179* |
|  | (0.00563) | (0.000968) | (0.00329) | (0.00353) | (0.0179) | (0.0449) | (0.0550) | (0.0148) | (0.0393) | (0.00577) | (0.108) |
|  |  |  |  |  |  |  |  |  |  |  |  |
| 18-30 years old | 0.957 | 0.986 | 1.057 | 0.991 | 1.320* | 0.883 | 1.003 | 1.079 | 1.241 | 1.394* | 1.502** |
|  | (0.151) | (0.201) | (0.188) | (0.177) | (0.197) | (0.135) | (0.161) | (0.164) | (0.230) | (0.245) | (0.277) |
|  |  |  |  |  |  |  |  |  |  |  |  |
| 31-40 years old | 1.049 | 0.936 | 1.410* | 0.921 | 1.451** | 1.278 | 1.401** | 1.265 | 1.556** | 1.769*** | 1.743*** |
|  | (0.172) | (0.213) | (0.291) | (0.166) | (0.229) | (0.200) | (0.230) | (0.207) | (0.306) | (0.350) | (0.335) |
|  |  |  |  |  |  |  |  |  |  |  |  |
| 41-50 years old | 0.931 | 0.926 | 1.212 | 0.931 | 1.459** | 1.185 | 1.169 | 1.312* | 1.402* | 1.396* | 1.553** |
|  | (0.147) | (0.194) | (0.217) | (0.162) | (0.223) | (0.183) | (0.192) | (0.203) | (0.279) | (0.256) | (0.300) |
|  |  |  |  |  |  |  |  |  |  |  |  |
| 51-60 years old | 1.056 | 0.804 | 1.091 | 0.855 | 1.587*** | 1.454** | 1.115 | 1.053 | 1.246 | 1.013 | 1.166 |
|  | (0.181) | (0.188) | (0.193) | (0.157) | (0.250) | (0.238) | (0.193) | (0.167) | (0.255) | (0.185) | (0.243) |
|  |  |  |  |  |  |  |  |  |  |  |  |
| Female | 1.103 | 1.084 | 1.011 | 1.140 | 1.135* | 1.183** | 1.163* | 1.104 | 1.061 | 0.975 | 1.125 |
|  | (0.0877) | (0.121) | (0.0889) | (0.0962) | (0.0820) | (0.0898) | (0.0922) | (0.0825) | (0.0938) | (0.0878) | (0.101) |
|  |  |  |  |  |  |  |  |  |  |  |  |
| Less than High School | 0.849 | 0.993 | 0.695*** | 0.743*** | 0.641*** | 0.579*** | 0.551*** | 0.591*** | 0.741*** | 0.545*** | 0.564*** |
|  | (0.0854) | (0.140) | (0.0804) | (0.0795) | (0.0585) | (0.0563) | (0.0563) | (0.0563) | (0.0855) | (0.0631) | (0.0648) |
|  |  |  |  |  |  |  |  |  |  |  |  |
| High School | 1.015 | 0.989 | 0.849* | 0.719*** | 0.781*** | 0.773*** | 0.721*** | 0.761*** | 0.753*** | 0.780** | 0.657*** |
|  | (0.0858) | (0.101) | (0.0758) | (0.0632) | (0.0605) | (0.0671) | (0.0678) | (0.0634) | (0.0750) | (0.0772) | (0.0676) |
|  |  |  |  |  |  |  |  |  |  |  |  |
| Child<12 present | 1.001 | 0.918 | 0.839* | 1.014 | 0.946 | 0.987 | 0.972 | 0.982 | 1.011 | 0.974 | 0.956 |
|  | (0.0842) | (0.100) | (0.0813) | (0.0867) | (0.0732) | (0.0787) | (0.0813) | (0.0798) | (0.0937) | (0.0938) | (0.0955) |
|  |  |  |  |  |  |  |  |  |  |  |  |
| Senior present | 0.947 | 0.894 | 0.993 | 0.894 | 0.993 | 0.939 | 1.027 | 0.990 | 0.972 | 1.036 | 1.081 |
|  | (0.0903) | (0.115) | (0.112) | (0.0895) | (0.0882) | (0.0877) | (0.0983) | (0.0904) | (0.0998) | (0.113) | (0.114) |
|  |  |  |  |  |  |  |  |  |  |  |  |
| Trust Gov. | 1.096 | 0.966 | 1.120 | 0.905 | 0.946 | 0.869 | 0.969 | 0.859* | 0.913 | 0.740*** | 0.784** |
|  | (0.102) | (0.125) | (0.110) | (0.0872) | (0.0765) | (0.0750) | (0.0851) | (0.0729) | (0.0901) | (0.0763) | (0.0795) |
|  |  |  |  |  |  |  |  |  |  |  |  |
| Trust others | 0.904 | 1.059 | 0.867 | 0.918 | 0.889 | 0.823** | 1.111 | 1.042 | 0.913 | 1.032 | 1.006 |
|  | (0.0892) | (0.132) | (0.0993) | (0.1000) | (0.0804) | (0.0805) | (0.119) | (0.103) | (0.107) | (0.116) | (0.130) |
|  |  |  |  |  |  |  |  |  |  |  |  |
| Observations | 7337 | 7191 | 7084 | 7070 | 7015 | 7309 | 6377 | 7084 | 4654 | 5276 | 4725 |
| FE by country | YES | YES | YES | YES | YES | YES | YES | YES | YES | YES | YES |
| Pearson Chi2 | 4710.26 | 4795.96 | 3948.44 | 3800.79 | 3768.08 | 3870.06 | 3556.09 | 3932.73 | 2748.84 | 3265.61 | 2952.94 |
| Hosmer-Lemeshow Chi2(8) | 575.97 | 52.96 | 80.54 | 211.78 | 68.18 | 4.81 | 21.93 | 105.62 | 8.77 | 76.61 | 19.32 |

* p<0.10 ** p<0.05 *** p<0.01

Note: In the case of “self” the dependent variable is an indicator variable taking the value 1 if a respondent claims to ‘Always” perform the behavior. In the case of “others” the dependent variable is an indicator variable taking the value 1 if the respondent state that others “Never” or “Sometimes” perform the behavior better than themselves. We use a Logit Regressions. Robust standard errors in parentheses. Population weights used. Odds Ratio displayed (a coefficient of 2 implies the odds that an individual complies with a preventive behavior is 2 times higher than for the base category). Base categories: 61 years old or more, more than HS (high school); men; no children under 12 at home; no seniors at home; no trust. Includes country FE.

Table F.1 shows the Odds Ratios of the models run in the main results section but performed through a Logit regression. Note that results hold. There is evidence of overconfidence in every preventive behavior except for keeping distance at the public transportation. Moreover, the largest effects are seen for using masks inside close stores and the lowest are for keeping the distance on the street and at the market. Nevertheless, our goodness of fit measures (the Pearson and Hosmer-Lemeshow goodness-of-fit test) show that the Logit model does not fit well: in every instant except for distance on the street and at the park, we reject our model.

**F.2 Robustness checks**

As a robustness test, we run a model comparing the proportion of individuals who state that they “Always” comply in the “*Self*” group with those that state that others “Always comply better than me” in the “*Others*” group. It is possible that individuals in the “*Others*” group did not properly understand the question, and instead of answering to “how much more frequent than you did the rest of your fellow citizens comply with the use of masks, hand wash or social distance”, they simply replied to how much they believe others comply with the preventive measure. In other words, they replied strictly thinking about the behavior of others rather than their own behavior compared to others. Therefore, given that that two samples are balanced, then it cannot happen that if 80 percent of the “*Self*” group says they always comply, then only 30 percent of the “*Others*” group says that the others always comply. As such, we would be in the presence of overconfidence if when we ask individuals about their own behavior, they say they comply at a higher rate than when asked about the behavior of others. Table F.2 shows the results. As it can be seen, there is evidence of overconfidence in every measure as it is always the case that asking about others give a lower compliance than when asked about individual’s own behavior. Therefore, even if respondents did not understand the question correctly, the conclusion stays the same.

**Table F.2 Robustness check: “Always comply” vs. “Others always comply”**

|  | (1) | (2) | (3) | (4) | (5) | (6) | (7) | (8) | (9) | (10) | (11) |
| --- | --- | --- | --- | --- | --- | --- | --- | --- | --- | --- | --- |
|  | Compliance with preventive behaviors | | | | | Compliance with distance | | | | | |
|  | Mask in public | Mask in store | Wash hand | Hand sanitizer in store | Sneeze on elbow | On the street | At the market | At the store | At the park | At health center | In public transport |
|  |  |  |  |  |  |  |  |  |  |  |  |
| Others (β1) | -0.380*** | -0.193*** | -0.263*** | -0.173*** | -0.328*** | -0.561*** | -0.550*** | -0.420*** | -0.511*** | -0.340*** | -0.413*** |
|  | (0.0118) | (0.0101) | (0.0121) | (0.0116) | (0.0143) | (0.0125) | (0.0134) | (0.0131) | (0.0150) | (0.0135) | (0.0184) |
|  |  |  |  |  |  |  |  |  |  |  |  |
| 18-30 years old | -0.0167 | 0.00366 | -0.0851*** | -0.0372 | 0.000224 | -0.102*** | -0.0208 | -0.0782*** | -0.151*** | -0.0506* | -0.0541 |
|  | (0.0241) | (0.0195) | (0.0242) | (0.0248) | (0.0312) | (0.0278) | (0.0284) | (0.0281) | (0.0346) | (0.0287) | (0.0386) |
|  |  |  |  |  |  |  |  |  |  |  |  |
| 31-40 years old | -0.00953 | 0.000448 | -0.0567** | -0.00383 | 0.0311 | -0.0774*** | -0.0385 | -0.0499* | -0.114*** | -0.0846*** | -0.0402 |
|  | (0.0246) | (0.0213) | (0.0272) | (0.0252) | (0.0320) | (0.0264) | (0.0279) | (0.0289) | (0.0358) | (0.0317) | (0.0394) |
|  |  |  |  |  |  |  |  |  |  |  |  |
| 41-50 years old | -0.00338 | 0.0182 | -0.0329 | 0.0256 | 0.0366 | -0.0568** | -0.0487* | -0.0311 | -0.138*** | -0.0466 | -0.0644 |
|  | (0.0234) | (0.0197) | (0.0235) | (0.0241) | (0.0312) | (0.0264) | (0.0285) | (0.0275) | (0.0362) | (0.0291) | (0.0400) |
|  |  |  |  |  |  |  |  |  |  |  |  |
| 51-60 years old | -0.0160 | 0.0200 | 0.00831 | 0.0439* | 0.0384 | -0.0215 | 0.0106 | 0.0181 | -0.0779** | 0.0216 | -0.0363 |
|  | (0.0248) | (0.0211) | (0.0232) | (0.0248) | (0.0321) | (0.0271) | (0.0302) | (0.0284) | (0.0380) | (0.0293) | (0.0442) |
|  |  |  |  |  |  |  |  |  |  |  |  |
| Female | 0.0210* | 0.00308 | 0.0272** | 0.0258** | 0.0189 | 0.0316** | 0.0106 | 0.0100 | 0.0237 | 0.0242* | 0.0247 |
|  | (0.0118) | (0.0102) | (0.0118) | (0.0116) | (0.0142) | (0.0126) | (0.0132) | (0.0132) | (0.0157) | (0.0145) | (0.0179) |
|  |  |  |  |  |  |  |  |  |  |  |  |
| Less than High School | 0.0217 | -0.0183 | 0.0154 | 0.0157 | 0.0490*** | 0.0508*** | 0.0726*** | 0.0732*** | 0.0331 | 0.0796*** | 0.0848*** |
|  | (0.0149) | (0.0128) | (0.0154) | (0.0149) | (0.0179) | (0.0157) | (0.0166) | (0.0168) | (0.0205) | (0.0187) | (0.0223) |
|  |  |  |  |  |  |  |  |  |  |  |  |
| High School | 0.00334 | 0.00508 | 0.00107 | 0.0225* | 0.00759 | -0.00382 | 0.00749 | 0.0355*** | 0.0137 | 0.0342** | 0.0362** |
|  | (0.0124) | (0.00926) | (0.0119) | (0.0120) | (0.0146) | (0.0127) | (0.0138) | (0.0137) | (0.0169) | (0.0153) | (0.0183) |
|  |  |  |  |  |  |  |  |  |  |  |  |
| Child<12 present | 0.00951 | 0.00596 | 0.0365*** | 0.0112 | 0.0210 | 0.0178 | 0.00664 | 0.0328** | 0.0202 | 0.0118 | 0.00995 |
|  | (0.0125) | (0.00981) | (0.0128) | (0.0118) | (0.0153) | (0.0131) | (0.0140) | (0.0143) | (0.0163) | (0.0153) | (0.0199) |
|  |  |  |  |  |  |  |  |  |  |  |  |
| Senior present | 0.0182 | 0.0138 | 0.0119 | 0.0116 | 0.0126 | -0.00294 | 0.00941 | 0.0225 | -0.0249 | 0.0211 | 0.00973 |
|  | (0.0146) | (0.0115) | (0.0153) | (0.0140) | (0.0172) | (0.0156) | (0.0157) | (0.0162) | (0.0181) | (0.0176) | (0.0208) |
|  |  |  |  |  |  |  |  |  |  |  |  |
| Trust Gov. | 0.0297** | 0.0208* | -0.00203 | 0.0108 | -0.000529 | 0.00834 | 0.0152 | 0.0427*** | 0.0383** | 0.0531*** | 0.0354* |
|  | (0.0138) | (0.0116) | (0.0134) | (0.0135) | (0.0160) | (0.0148) | (0.0151) | (0.0150) | (0.0178) | (0.0165) | (0.0202) |
|  |  |  |  |  |  |  |  |  |  |  |  |
| Trust others | 0.00755 | -0.00591 | -0.0142 | 0.00591 | 0.00735 | 0.0221 | 0.0222 | -0.0154 | -0.00146 | 0.0136 | 0.0457* |
|  | (0.0150) | (0.0119) | (0.0154) | (0.0146) | (0.0187) | (0.0168) | (0.0171) | (0.0171) | (0.0210) | (0.0179) | (0.0250) |
|  |  |  |  |  |  |  |  |  |  |  |  |
| Constant | 0.973*** | 0.963*** | 0.911*** | 0.875*** | 0.730*** | 0.917*** | 0.848*** | 0.807*** | 0.941*** | 0.864*** | 0.719*** |
|  | (0.0368) | (0.0328) | (0.0384) | (0.0397) | (0.0437) | (0.0380) | (0.0375) | (0.0393) | (0.0466) | (0.0421) | (0.0534) |
|  |  |  |  |  |  |  |  |  |  |  |  |
| Observations | 7337 | 7191 | 7084 | 7070 | 7015 | 7309 | 6377 | 7084 | 4654 | 5276 | 4725 |
| FE by country | YES | YES | YES | YES | YES | YES | YES | YES | YES | YES | YES |
| R-squared | 0.263 | 0.124 | 0.149 | 0.114 | 0.152 | 0.353 | 0.339 | 0.239 | 0.292 | 0.183 | 0.204 |

* p<0.10 ** p<0.05 *** p<0.01

Note: In the case of “Self” the dependent variable is an indicator variable taking the value 1 if a respondent claims to ‘Always” perform the behavior. In the case of “Others” the dependent variable is an indicator variable taking the value 1 if the respondent state that others “Never” or “Sometimes” perform the behavior better than themselves. We use a Linear Probability Model. Robust standard errors in parentheses. Population weights used. Marginal effects displayed (a coefficient of 0.1 implies it is 10 p.p. more likely to comply with the preventive behavior the base category). Base categories: "Self" group, 61 years old or more, more than HS (high school); men; no children under 12 at home; no seniors at home; no trust. Includes country FE.

Second, given that there might be social desirability bias, the rate of compliance stated by those asked about their own behavior might not reflect the true distribution in the population. For that reason, we perform the same estimation but changing the outcome. Particularly we use five questions related to support for different governmental surveillance methods to guarantee that those COVID-19 positive in fact quarantine (telephone checks, home visits, electronic bracelets, track through GPS on cellphone, guard at home). For the group that was asked about own behavior, the questions asked about general support of such methods. For the group that was asked about behavior relative to others, the question asked about support considering they were a COVID-19 positive case themselves. Particularly:

- ***Others****: People with coronavirus should isolate themselves so as not to spread and stay in quarantine. To verify that all patients are kept in quarantine, do you support or not support that government authorities ...?*
- ***Self****: People with coronavirus should isolate themselves so as not to spread and stay in quarantine. If you were a positive case of coronavirus, do you support or do not support that to corroborate that you are in quarantine government authorities ...?*

According to Wise et al. [17], feeling personally at risk is the most important predictor for engaging in preventive behaviors in the context of COVID-19. Then, the rate at which individuals support different surveillance methods should be higher if the one under quarantine is a random person than if it is oneself: the risk of no surveillance for the first means an increased probability of exposure for the respondent, while the second is a strict control over one’s own movements. Nevertheless, the only surveillance method for which that happens is telephone checks (Column (1) of Table F.3). For the rest of the more intrusive surveillance methods, we do not observe significant differences in support between the groups. Therefore, we can conclude that social desirability bias is not driving our main results, given that there are no differences for support on surveillance methods if the one under quarantine is a random person than if it is the individual who is being surveyed.

**Table F.3. Robustness check: alternative outcomes**

|  | (1) | (2) | (3) | (4) | (5) |
| --- | --- | --- | --- | --- | --- |
|  | Support for surveillance methods | | | | |
|  | Telephone | Visits | Electronic Bracelet | GPS | Guard |
|  |  |  |  |  |  |
| Others (β1) | 0.0230** | 0.0226 | 0.0213 | -0.0000227 | 0.00137 |
|  | (0.0103) | (0.0140) | (0.0155) | (0.0145) | (0.0147) |
|  |  |  |  |  |  |
| 18-30 years old | 0.0423** | -0.0566** | 0.0994*** | -0.0497* | 0.0340 |
|  | (0.0174) | (0.0268) | (0.0298) | (0.0275) | (0.0294) |
|  |  |  |  |  |  |
| 31-40 years old | 0.0182 | -0.00989 | 0.0271 | -0.0463 | -0.0393 |
|  | (0.0193) | (0.0280) | (0.0320) | (0.0284) | (0.0306) |
|  |  |  |  |  |  |
| 41-50 years old | 0.00777 | 0.00538 | 0.0236 | -0.0328 | -0.0565* |
|  | (0.0185) | (0.0265) | (0.0304) | (0.0273) | (0.0294) |
|  |  |  |  |  |  |
| 51-60 years old | 0.00845 | 0.0422 | 0.0290 | 0.0275 | -0.0158 |
|  | (0.0215) | (0.0279) | (0.0329) | (0.0287) | (0.0315) |
|  |  |  |  |  |  |
| Female | 0.00192 | -0.0583*** | -0.0291* | 0.0198 | 0.00234 |
|  | (0.00997) | (0.0139) | (0.0153) | (0.0143) | (0.0145) |
|  |  |  |  |  |  |
| Less than High School | 0.0142 | 0.0612*** | 0.0729*** | 0.0429** | 0.226*** |
|  | (0.0122) | (0.0180) | (0.0193) | (0.0183) | (0.0178) |
|  |  |  |  |  |  |
| High School | -0.0173 | 0.0554*** | 0.0195 | 0.00250 | 0.0860*** |
|  | (0.0111) | (0.0151) | (0.0160) | (0.0153) | (0.0143) |
|  |  |  |  |  |  |
| Child<12 present | 0.00749 | 0.0110 | 0.00209 | 0.00663 | 0.0289* |
|  | (0.0119) | (0.0151) | (0.0167) | (0.0156) | (0.0161) |
|  |  |  |  |  |  |
| Senior present | 0.0101 | 0.0186 | 0.00770 | -0.00246 | -0.00792 |
|  | (0.0111) | (0.0168) | (0.0183) | (0.0170) | (0.0178) |
|  |  |  |  |  |  |
| Trust Gov. | 0.0649*** | 0.0329** | 0.0515*** | 0.0739*** | 0.0198 |
|  | (0.0113) | (0.0156) | (0.0171) | (0.0161) | (0.0162) |
|  |  |  |  |  |  |
| Trust others | -0.00194 | 0.00140 | -0.0293 | -0.0375* | -0.0297 |
|  | (0.0154) | (0.0193) | (0.0214) | (0.0203) | (0.0202) |
|  |  |  |  |  |  |
| Constant | 0.834*** | 0.770*** | 0.502*** | 0.695*** | 0.173*** |
|  | (0.0241) | (0.0384) | (0.0460) | (0.0422) | (0.0413) |
|  |  |  |  |  |  |
| Observations | 7337 | 7191 | 7084 | 7070 | 7015 |
| FE by country | YES | YES | YES | YES | YES |
| R-squared | 0.023 | 0.070 | 0.049 | 0.035 | 0.093 |

Note: Results of Linear Probability Model. Robust standard errors in parenthesis. Population weights used.* p<0.10 ** p<0.05 *** p<0.01. Marginal effects displayed (a coefficient of 0.1 implies it is 10 p.p. more likely to comply with the preventive behavior the base category). Base categories: belonging to the "Self" group, 61 years old or more, more than HS (high school); men; no children under 12 at home; no seniors at home; no trust. Includes country FE.

**G Instrument (Spanish)**

**Buenas tardes, mi nombre es ____________ y trabajo para FIRMA, una empresa que hace estudios de opinión pública.**

**Estamos haciendo una encuesta, en el marco de un estudio internacional sobre la emergencia en salud a causa del coronavirus y las herramientas para disminuir el contagio, entre ellas el uso de tecnología. La encuesta dura alrededor de 15 minutos. Sus respuestas son voluntarias y estrictamente confidenciales**. **¿Accede a participar?**

**P0. VERSION CUESTIONARIO**

**1. CUESTIONARIO 1**

**2. CUESTIONARIO 2**

**3. CUESTIONARIO 3**

**4. CUESTIONARIO 4**

**P1. ¿Cuántos años tiene usted?**

________________________________

**P2. (ANOTAR GENERO POR REGISTRO DE VOZ o preguntar si hay dudas)**

1. Hombre
2. Mujer

**P3. ¿Cuál es el nivel educativo mas alto que alcanzó? (respuesta abierta, encuestador anota opción más cercana**. Como referencia: Primaria = entre 6 y 11 años; Secundaria = entre 12 y 18 años)

- 1. No fue a la escuela
  2. Primaria incompleta
  3. Primaria completa
  4. Secundaria incompleta
  5. Secundaria completa
  6. Universitario/terciario incompleto
  7. Universitario/terciario completa
  8. Posgrado incompleto
  9. Posgrado completo

99. Ns/Nr

**Modulo 2: Hogar**

**P4. Incluido usted, ¿cuántas personas residen en el hogar?**

________________________________

**P5. Sin contarse usted, ¿Hay mayores de 60 años en el hogar?**

1. Sí
2. No

99. NS/NR

**P6. ¿Hay menores de 12 años en el hogar?**

1. Sí
2. No

99. NS/NR

**Modulo 3: Uso de Tecnología**

**P7. ¿Utilizó en la última semana un teléfono inteligente, conocido también como “Smartphone”?**

1. Sí
2. No

99. NS/NR

**P8. (P7 =1) La semana pasada, ¿cuántas veces realizó las siguientes actividades con su celular o en internet de cualquier forma? ¿todos los días, algunos días, o nunca realizó? Ns/Nr=99. (Lea las alternativas y marque una respuesta para cada ítem).**

| **Actividad** | **1 –**  **Todos los días** | **2 – Algunos días** | **3 - Nunca** | **99 – Ns/Nr** |
| --- | --- | --- | --- | --- |
| P8_1 Uso de redes sociales (Facebook, Instagram, Twitter, etc.) |  |  |  |  |
| P8_2 Uso de mensajería instantánea (WhatsApp, iMessage, Messenger, etc.) |  |  |  |  |
| P8_3 Hacer compras o pagar servicios online |  |  |  |  |

**Modulo 4: Confianza y Coronavirus**

**P9. De las siguientes dos frases, ¿con cuál se identifica más? (i) se puede confiar en la mayoría de las personas; o (ii) uno nunca es lo suficientemente cuidadoso en el trato con los demás**

1. Se puede confiar en la mayoría de las personas

2. Uno nunca es lo suficientemente cuidadoso en el trato con los demás

99. NS/NR

**P10. ¿Cuánta confianza tiene usted en el gobierno? ¿Mucha, algo, o nada?**

1. Mucha

2. Algo

3. Nada

99. NS/NR

**P11. Pensando en la información que recibe sobre el coronavirus, ¿usted tiene mucha confianza en la información que da el presidente, algo, o ninguna confianza? ¿Y en…?**

| **Institución** | **1-Mucha** | **2-Algo** | **3-Ninguna** | **77. No recibe** | **99-Ns/Nr** |
| --- | --- | --- | --- | --- | --- |
| P11_1 Presidente |  |  |  |  |  |
| P11_2 (AUTORIDAD LOCAL/Gobernador, Intendente) |  |  |  |  |  |
| P11_3 Medios de comunicación (diarios, radio, televisión) |  |  |  |  |  |
| P11_4 Posteos en redes sociales (Twitter, Facebook, etc.) |  |  |  |  |  |
| P11_5 Organización Mundial de la Salud |  |  |  |  |  |
| P11_6 Ministro de Salud |  |  |  |  |  |

**P12A. (P0=1 O 2) Las personas con coronavirus deben aislarse para no contagiar y mantenerse en cuarentena. Para corroborar que todos los enfermos se mantengan en cuarentena, ¿Usted apoya o no apoya que autoridades del gobierno…?**

| **Normas de monitoreo** | **1-Apoya** | **2-No apoya** | **99-Ns/Nr** |
| --- | --- | --- | --- |
| P12A_1 Llamen por teléfono todos los días a las personas con coronavirus |  |  |  |
| P12A_2 Visiten todos los días a las personas con coronavirus |  |  |  |
| P12A_3 Les pongan pulseras electrónicas a las personas con coronavirus |  |  |  |
| P12A_4 Rastreen dónde están los enfermos con el teléfono celular (vía sistema de GPS) |  |  |  |
| P12A_5 Pongan un guardia en la puerta del lugar donde está el enfermo |  |  |  |

**P12b. (P0=3 O 4) Las personas con coronavirus deben aislarse para no contagiar y mantenerse en cuarentena. Si usted fuese un caso positivo de coronavirus, ¿Usted apoya o no apoya que para corroborar que usted se mantenga en cuarentena autoridades del gobierno…?**

| **Normas de monitoreo** | **1-Apoya** | **2-No apoya** | **99-Ns/Nr** |
| --- | --- | --- | --- |
| P12C_1 Llamen por teléfono todos los días a las personas con coronavirus |  |  |  |
| P12C_2 Visiten todos los días a las personas con coronavirus |  |  |  |
| P12C_3 Les pongan pulseras electrónicas a las personas con coronavirus |  |  |  |
| P12C_4 Rastreen dónde están los enfermos con el teléfono celular (vía sistema de GPS) |  |  |  |
| P12C_5 Pongan un guardia en la puerta del lugar donde está el enfermo |  |  |  |

**Modulo 5: Trámites**

**P13. Entre enero y marzo, antes de que hubiera restricciones al movimiento (llamado “cuarentena” en algunos lugares), ¿hizo algún trámite con una institución de gobierno, como renovar documento de identidad, licencia de conducir, acceder a seguro de desempleo, o cualquier otro trámite? Puede ser para su vida personal o para su trabajo, y por cualquier canal de atención (en persona, en línea, etc.).**

1. Sí
2. No

99. NS/NR

**P14. (P13=1) ¿Qué tipo de trámite fue? Si hizo más de uno, queremos saber sobre el último trámite que hizo. (respuesta abierta, encuestador anota opción más cercana)**

1. Solicitar o renovar un documento de identidad o registro civil
2. Acceder a un programa social, o tramitar seguro de desempleo
3. Acceder a un servicio de educación o salud
4. Registrar, comprar o vender una propiedad inmueble
5. Abrir o cerrar una empresa, u otro trámite empresarial
6. Pagar impuestos, pagar seguro médico, pagar o cobrar una pensión/mesada pública por jubilación
7. Hacer una denuncia de un crimen
8. Solicitar un permiso de conducir u otro trámite de transporte
9. Un trámite de justicia (por ejemplo, consulta a mi juicio)
10. Otro (ANOTAR)

**P15. (P13=1) Para el mismo trámite que nos acaba de señalar, ¿Fue personalmente a una oficina pública, hizo el trámite por internet, hizo el trámite por teléfono, o empezó el trámite por internet o por teléfono y lo terminó personalmente?**

1. Todo presencial / en la oficina pública
2. Una parte por teléfono y otra en la oficina
3. Una parte en línea y otra en la oficina
4. Todo por teléfono
5. Todo en línea

99. NS/NR

**P16. Durante el periodo de mayores restricciones al movimiento (llamado “cuarentena” en algunos lugares), ¿hizo algún trámite con una institución de gobierno, como renovar documento de identidad, licencia de conducir, acceder a seguro de desempleo, o cualquier otro trámite? Puede ser para su vida personal o para su trabajo, y por cualquier canal de atención (en persona, en línea, etc.).**

1. Sí
2. No

99. NS/NR

**P17. (P16=1) Si hizo más de un trámite durante el periodo de mayores restricciones al movimiento, queremos saber sobre el trámite más reciente. ¿Qué tipo de trámite fue? (respuesta abierta, encuestador anota opción más cercana)**

1. Solicitar o renovar un documento de identidad o registro civil
2. Acceder a un programa social, tramitar seguro de desempleo
3. Acceder a un servicio de educación o salud
4. Registrar, comprar o vender una propiedad inmueble
5. Abrir o cerrar una empresa, u otro trámite empresarial
6. Pagar impuestos, pagar seguro médico, pagar o cobrar una pensión/mesada pública por jubilación
7. Hacer una denuncia de un crimen
8. Solicitar un permiso de conducir u otro trámite de transporte
9. Un trámite de justicia (por ejemplo, consulta a mi juicio)
10. Otro (ANOTAR)

**P18. (P16=1) ¿Fue personalmente a una oficina pública, hizo el trámite por internet, hizo el trámite por teléfono, o empezó el trámite por internet y lo terminó personalmente?**

1. Todo presencial / la oficina pública
2. Una parte por teléfono y otra en la oficina
3. Una parte en línea y otra en la oficina
4. Todo por teléfono
5. Todo en línea

99. NS/NR

**P19. (P18=1, 2 o 3) ¿Considera usted que se respetaron las normas de distanciamiento social y seguridad sanitaria en su visita a la oficina prestadora?**

1. Sí

2. No

99. NS/NR

**P20. Durante el periodo de cuarentena más restrictiva, ¿hubo algún trámite oficial que hubiera querido hacer pero que no pudo? (para su vida personal o para su trabajo)**

1. Sí

2. No

99. NS/NR

**P21. (P20=1) Pensando en el trámite MAS IMPORTANTE que no pudo hacer, ¿Qué tipo de trámite fue?** (respuesta abierta, encuestador anota opción más cercana)

1. Solicitar o renovar un documento de identidad o registro civil
2. Acceder a un programa social, tramitar seguro de desempleo
3. Acceder a un servicio de educación o salud
4. Registrar, comprar o vender una propiedad inmueble
5. Abrir o cerrar una empresa, u otro trámite empresarial
6. Pagar impuestos, pagar seguro médico, pagar o cobrar una pensión/mesada pública por jubilación
7. Hacer una denuncia de un crimen
8. Solicitar un permiso de conducir u otro trámite de transporte
9. Un trámite de justicia (por ejemplo, consulta a mi juicio)
10. Otro (ANOTAR)

**P22. (P20=1) ¿Por qué no lo pudo hacer?** (respuesta abierta, encuestador anota opción más cercana)

1. La oficina que presta el trámite estaba abierta, pero no quise ir en persona
2. No atendían el teléfono, mucha espera para que atendieran
3. Se cerró la oficina pública donde se presta y no estaba disponible en línea
4. El trámite que buscaba estaba disponible en línea, pero no lo pude completar (porque no tenía un computador o dispositivo, el trámite no se pudo hacer o no se completó).
5. Otra razón (ANOTAR)

**Modulo 6: Tecnología y coronavirus**

SOLO LOS QUE TIENEN TELEFONO INTELIGENTE

**P23A. (PO=1 O 3) (P7=1) Si existe o hubiera una aplicación del gobierno nacional que usted necesitaría descargar (pero que no le consumiría datos ni saldo) que le permite saber si tiene algún síntoma de coronavirus y le diga qué hacer, ¿seguramente la instalaría en su teléfono, probablemente la instalaría, o no la instalaría?**

1. (Espontánea) Ya instaló/tiene la oficial del país

2. Seguro la instalaría

3. Probablemente la instalaría

4. No la instalaría

99. NS/NR

**P23B. (P0=2 O 4) (P7=1) Si existe o hubiera una aplicación del gobierno nacional que se instalaría automáticamente con posibilidad de desinstalar cuando desee (pero que no le consumiría datos ni saldo) que le permite saber si tiene algún síntoma de coronavirus y le diga qué hacer, ¿seguramente la desinstalaría en su teléfono, probablemente, o no la desinstalaría?**

1. (Espontánea) Ya instaló/tiene la oficial del país

2. Seguro la desinstalaría

3. Probablemente la desinstalaría

4. No la desinstalaría

99. NS/NR

**P24A. (P0=1 O 3) (P7=1) Si esa aplicación también le alertara si usted estuvo en contacto por más de 15 minutos con una persona infectada de coronavirus y le notificara a las personas que estuvieron en contacto cercano con usted, sin identificar ningún nombre, ni el suyo ni el de las otras personas, ¿seguramente la instalaría en su teléfono, probablemente la instalaría, o no la instalaría?**

1. (Espontánea) Ya instaló/tiene la oficial del país

2. Seguro la instalaría

3. Probablemente la instalaría

4. No la instalaría

99. NS/NR

**P24B. (P0=2 O 4) (P7=1) Si esa aplicación también le alertara si usted estuvo en contacto por más de 15 minutos con una persona infectada de coronavirus y le notificara a las personas que estuvieron en contacto cercano con usted, sin identificar ningún nombre, ni el suyo ni el de las otras personas, ¿seguramente la desinstalaría en su teléfono, probablemente la desinstalaría, o no la desinstalaría?**

1. (Espontánea) Ya instaló/tiene la oficial del país

2. Seguro la desinstalaría

3. Probablemente la desinstalaría

4. No la desinstalaría

99. NS/NR

**P25A. (P0=1 O 3)** (P23A=1, 2, o 3 o P24A=1, 2 o 3-LA INSTALÓ O LA INSTALARIA) **¿Cuáles serían las principales razones para instalar la aplicación?** (Sin leer las alternativas, marque lo que dice la gente) OPCION MULTIPLE

P25A_1 Saber mi nivel de riesgo de estar infectado

P25A_2 Mantenerme saludable

P25A_3 Proteger a mi familia

P25A_4 Mantenerme informado

P25A_5 Paz mental de saber que no estoy en riesgo

P25A_6 Responsabilidad ante la comunidad

P25A_7 Reducir el número de fallecimientos entre la gente mayor

P25A_8 Puede ayudar a detener la pandemia

P25A_9 Otro: _______

**P25B. (P0=2 O 4)**  (P23B=1 o 4 o P24B=1 o 4 LA INSTALÓ O NO LA DESINSTALARÍA) **¿Cuáles serían las principales razones para NO desinstalar la aplicación?** (Sin leer las alternativas, marque lo que dice la gente) OPCION MULTIPLE

P25B_1 Saber mi nivel de riesgo de estar infectado

P25B_2 Mantenerme saludable

P25B_3 Proteger a mi familia

P25B_4 Mantenerme informado

P25B_5 Paz mental de saber que no estoy en riesgo

P25B_6 Responsabilidad ante la comunidad

P25B_7 Reducir el número de fallecimientos entre la gente mayor

P25B_8 Puede ayudar a detener la pandemia

P25B_9 Otro: _______

**P26A. (P0=1 O 3)**  (P23=4, 99 y P24=4, 99 NO LA INSTALARIA O NO SABE**) ¿Cuáles son las principales razones para NO instalar la aplicación?** (Sin leer las alternativas, marque lo que dice la gente) OPCION MULTIPLE

P26A_1 No creo que ayude a detener la epidemia

P26A_2 No sé instalar aplicaciones

P26A_3 Sería muy complicado / no tengo espacio para instalarlo en mi teléfono

P26A_4 No creo beneficiarme de la aplicación

P26A_5 Me preocupa que mi teléfono pueda estar en peligro

P26A_6 Me preocupa que el gobierno utilice la aplicación como una excusa para tener más control sobre la ciudadanía una vez pase la pandemia

P26A_7 No quiero estar más ansioso de lo que ya estoy ahora

P26A_8 No quiero que el gobierno tenga acceso a mi locación

P26A_9 No creo en las promesas de anonimatos de las aplicaciones

P26A_10 Otro: _______

**P26B. (P0=2 O 4)** (P23B=2, 3 o 99 y P24B=2, 3 o 99 LA DESINSTALARIA O NO SABE) **¿Cuáles son las principales razones para desinstalar la aplicación?** (Sin leer las alternativas, marque lo que dice la gente) OPCION MULTIPLE

P26B_1 No creo que ayude a detener la epidemia

P26B_2 No sé desinstalar aplicaciones

P26B_3 Sería muy complicado / no tengo espacio para instalarlo en mi teléfono

P26B_4 No creo beneficiarme de la aplicación

P26B_5 Me preocupa que mi teléfono pueda estar en peligro

P26B_6 Me preocupa que el gobierno utilice la aplicación como una excusa para tener más control sobre la ciudadanía una vez pase la pandemia

P26B_7 No quiero estar más ansioso de lo que ya estoy ahora

P26B_8 No quiero que el gobierno tenga acceso a mi locación

P26B_9 No creo en las promesas de anonimatos de las aplicaciones

P26B_10 Otro: _______

**P27A. (P0=1 O 3)** P7=1 **¿Ud. seguro** **instalaría, probablemente instalaría o no instalaría la aplicación en su teléfono si…?**

| **Ítem** | **1. Seguro instalaría** | **2. Probablemente instalaría** | **3. No instalaría** | **99. NS/NR** |
| --- | --- | --- | --- | --- |
| P27A_1 Si usted fuese un caso positivo de coronavirus |  |  |  |  |
| P27A_2 Si alguien de su familia se encontrara infectado |  |  |  |  |
| P27A_3 Si la aplicación le permitiera obtener beneficios como descuentos en tiendas |  |  |  |  |

**P27B. (P0=2 O 4)** P7=1 **¿Ud. seguro desinstalaría, probablemente desinstalaría o no desinstalaría la aplicación en su teléfono si…?**

| **Ítem** | **1. Seguro desinstalaría** | **2. Probablemente desinstalaría** | **3. No desinstalaría** | **99. NS/NR** |
| --- | --- | --- | --- | --- |
| P27B_1 Si usted fuese un caso positivo de coronavirus |  |  |  |  |
| P27B_2 Si alguien de su familia se encontrara infectado |  |  |  |  |
| P27B_3 Si la aplicación le permitiera obtener beneficios como descuentos en tiendas |  |  |  |  |

**P28A.** **(P0=1 O 3)** P7=1 **Si en lugar de ser diseñada por el gobierno nacional la aplicación fuera diseñada por el gobierno local, ¿usted seguramente la instalaría, probablemente la instalaría o no la instalaría? ¿Y si fuera diseñada por…?**

| **Institución** | **1. Seguro instalaría** | **2. Probablemente instalaría** | **3. No instalaría** | **99. NS/NR** |
| --- | --- | --- | --- | --- |
| P28A_1 El gobierno local |  |  |  |  |
| P28A_2 Una compañía tecnológica internacional (Apple, Google, etc.) |  |  |  |  |
| P28A_3 Una compañía telefónica |  |  |  |  |
| P28A_4 La Organización Mundial de la Salud |  |  |  |  |

**P28B**. **(P0=2 O 4)** P7=1 **Si en lugar de ser diseñada por el gobierno nacional la aplicación fuera diseñada por el gobierno local, ¿usted seguramente la desinstalaría, probablemente la desinstalaría o no la desinstalaría? ¿Y si fuera diseñada por…?**

| **Institución** | **1. Seguro desinstalaría** | **2. Probablemente desinstalaría** | **3. No desinstalaría** | **99. NS/NR** |
| --- | --- | --- | --- | --- |
| P28B_1 El gobierno local |  |  |  |  |
| P28B_2 Una compañía tecnológica internacional (Apple, Google, etc.) |  |  |  |  |
| P28B_3 Una compañía telefónica |  |  |  |  |
| P28B_4 La Organización Mundial de la Salud |  |  |  |  |

**Modulo 7: confianza, tecnología y coronavirus**

**TODOS**

Ahora vamos a hacerle una serie de preguntas sobre la privacidad y protección de sus datos personales. Por datos personales, entendemos la información confidencial, como por ejemplo su historia clínica.

**P29. De acuerdo con lo que usted sabe o escuchó, ¿sus datos personales legalmente pueden ser utilizados por el gobierno en casos de emergencia?**

1. Sí

2. No

99. NS/NR

**P30. Si considera que hubo un mal uso de sus datos personales, ¿dónde lo denunciaría? (NO MENCIONAR OPCIONES DE RESPUESTA-LA OPCION 1. ES ESPECIFICA A CADA PAIS Y LAS DEMAS PARA TODOS)**

1. **Chile:** Consejo para la Transparencia

**Paraguay:** Cualquier ministerio / la oficina de acceso a la información pública dentro de cualquier ministerio

**Perú:** Autoridad Nacional de Protección de Datos Personales

**Uruguay:** Unidad Reguladora y de Control de Datos Personales / AGESIC / organismo de gobierno electrónico

1. defensor del pueblo/defensoría del pueblo/ombudsperson
2. fiscalía/ministerio de justicia
3. agencia de protección del consumidor, defensa del consumidor
4. agencia de acceso a la información
5. en la policía
6. en un banco u otra institución privada
7. no hay en dónde / no se puede
8. No sabe dónde
9. otro (ANOTAR)

99. NR

**P31. ¿Le parece que compartir sus datos personales tiene más beneficios que riesgos o más riesgos que beneficios?**

1. Más beneficios

2. Más riesgos

3. Depende con quién

99. NS/NR

**P32. ¿Ud. considera que tiene control sobre sus datos personales?**

1. Sí

2. Más o menos

3. No

99. NS/NR

**P33. ¿Usted sabe qué hacen las empresas con sus datos personales?**

1. Sí

2. Más o menos

3. No

99. NS/NR

**P34. ¿Usted sabe qué hace el gobierno con sus datos personales?**

1. Sí

2. Más o menos

3. No

99. NS/NR

**P35. ¿Qué es lo que más le preocupa sobre el uso que puedan hacer otros de sus datos personales?** (Respuesta abierta – encuestador anota respuesta más cercana)

1. Robo de identidad
2. Venta a un tercero
3. Uso para discriminación de parte de alguna entidad de gobierno
4. Uso para discriminación de parte de una empresa privada
5. Mercadeo no deseado
6. Estafas, robo de datos de tarjeta/cuenta bancaria
7. No me preocupa compartir mis datos personales
8. Invasión de la privacidad
9. Otro (ABIERTO, ANOTAR)

**P36. ¿Piensa que…?** (LEER CADA FRASE Y ANOTAR RESPUESTA)

| Frase | 1-  Sí | 2-  No | 99-  Ns/Nr |
| --- | --- | --- | --- |
| P36_1 **Los beneficios que el gobierno puede proporcionar al recopilar mis datos y el de otras personas superan los riesgos potenciales en épocas de pandemia?** |  |  |  |
| P36_2 **Para limitar la propagación del Covid 19 es imprescindible que el gobierno pueda rastrear los movimientos de todos nosotros para asegurarse que la gente limite el contacto social?** |  |  |  |
| P36_3 **El gobierno debería multar a las personas que son coronavirus positivo y no permiten la geo localización de sus celulares?** |  |  |  |

**Modulo 8: Comportamiento**

**P37. ¿Ud. ha escuchado sobre aplicaciones en su teléfono o sitios de web que le permitan reportar sus síntomas, sin ver un doctor, por ejemplo (EN URUGUAY coronavirus.uy)?**

1. Sí

2. No

**P38A. (P0=1 O 2)¿Cumple usted las recomendaciones de las autoridades de PAIS para prevenir el contagio del coronavirus?** (Lea las alternativas y marque una opción)

1. No cumplo ninguna
2. Cumplo alguna recomendación, alguna vez
3. Cumplo la mitad de las cosas que recomiendan, o la mitad de las veces
4. Cumplo la mayoría de las recomendaciones, la mayoría de las veces
5. Cumplo todas las recomendaciones, siempre

99. NS/NR

**P38B. (P0=3 O 4 ¿Cuánto MEJOR que usted cumplen el resto de los nacionalidad las recomendaciones de las autoridades de PAIS para prevenir el contagio del coronavirus? (Lea las alternativas y marque una opción)**

1. No cumplen ninguna mejor que yo
2. Cumplen alguna recomendación, alguna vez mejor que yo
3. Cumplen la mitad de las cosas que recomiendan, o la mitad de las veces mejor que yo
4. Cumplen la mayoría de las recomendaciones, la mayoría de las veces mejor que yo
5. Cumplen todas las recomendaciones, siempre mejor que yo

99. NS/NR

**P39. En la semana pasada, ¿usted salió de su hogar alguna vez?**

1. Sí
2. No

**P39A. (P0=1 O 2) y P39=1En la semana pasada, ¿ Con cuánta frecuencia cumplió con las siguientes recomendaciones? ¿Siempre, algunas veces, o nunca?**

| **Comportamiento** | **1**  **Siempre** | **2**  **Algunas veces** | **3**  **Nunca** | **98**  **No salió, No corresponde** | **99**  **Ns/**  **Nr** |
| --- | --- | --- | --- | --- | --- |
| **P39A_1** Tapabocas en la vía pública |  |  |  |  |  |
| **P39A_2** Tapabocas dentro de establecimientos (banco, supermercado, etc.) |  |  |  |  |  |
| **P39A_3** Tapabocas en su lugar de trabajo |  |  |  |  |  |
| **P39A_4** Lavarse las manos al volver a su hogar |  |  |  |  |  |
| **P39A_5** Alcohol en gel dentro de establecimientos (banco, comercio) |  |  |  |  |  |
| **P39A_6** Codo/antebrazo al toser/estornudar |  |  |  |  |  |

**P39B. (P0=3 O 4) y P39=1 En la semana pasada, ¿ Con cuánta MÁS frecuencia que usted cumplieron el resto de los nacionalidad con las siguientes recomendaciones? ¿Siempre, algunas veces, o nunca?**

| **Comportamiento** | **1**  **Siempre** | **2**  **Algunas veces** | **3**  **Nunca** | **98**  **No salió, No corresponde** | **99**  **Ns/**  **Nr** |
| --- | --- | --- | --- | --- | --- |
| **P39B_1** Tapabocas en la vía pública |  |  |  |  |  |
| **P39B_2** Tapabocas dentro de establecimientos (banco, supermercado, etc.) |  |  |  |  |  |
| **P39B_3** Tapabocas en su lugar de trabajo |  |  |  |  |  |
| **P39B_4** Lavarse las manos al volver a su hogar |  |  |  |  |  |
| **P39B_5** Alcohol en gel dentro de establecimientos (banco, comercio) |  |  |  |  |  |
| **P39B_6** Codo/antebrazo al toser/estornudar |  |  |  |  |  |

**P40A. (P0=1 O 2) De acuerdo a su percepción, ¿usted se preocupa por mantener la distancia de 2 metros en…? ¿Siempre, algunas veces, o nunca?**

| **Lugar** | **1**  **Siempre** | **2**  **Algunas veces** | **3**  **Nunca** | **98**  **No salió, no corresponde** | **99**  **NS/NR** |
| --- | --- | --- | --- | --- | --- |
| P40A_1 La vía pública |  |  |  |  |  |
| P40A_2 La feria / el mercado |  |  |  |  |  |
| P40A_3 Un local cerrado (supermercado, banco, etc.) |  |  |  |  |  |
| P40A_4 El parque |  |  |  |  |  |
| P40A_5 El centro de salud |  |  |  |  |  |
| P40A_6 El transporte público |  |  |  |  |  |

**P40B. (P0=3 O 4) De acuerdo a su percepción, ¿con cuánta MÁS frecuencia que usted se preocupan el resto de los nacionalidad por mantener la distancia de 2 metros en…? ¿Siempre, algunas veces, o nunca?**

| **Lugar** | **1**  **Siempre** | **2**  **Algunas veces** | **3**  **Nunca** | **98**  **No salió, no corresponde** | **99**  **NS/NR** |
| --- | --- | --- | --- | --- | --- |
| P40B_1 La vía pública |  |  |  |  |  |
| P40B_2 La feria / el mercado |  |  |  |  |  |
| P40B_3 Un local cerrado (supermercado, banco, etc.) |  |  |  |  |  |
| P40B_4 El parque |  |  |  |  |  |
| P40B_5 El centro de salud |  |  |  |  |  |
| P40B_6 El transporte público |  |  |  |  |  |

**P41. ¿Piensa que hasta que se desarrolle una vacuna las clases presenciales deberían estar prohibidas…**

| **Frase** | **1-Sí** | **2-**  **No** | **99-**  **Ns/Nr** |
| --- | --- | --- | --- |
| P41_1 para niños entre 0-5 años? |  |  |  |
| P41_2 para niños entre 6-12 años? |  |  |  |

AGRADECER Y FINALIZAR

P42. COMENTARIOS (ANOTA ENCUESTADOR CUALQUIER DUDA, AGREGADO QUE QUIERA REALIZAR A LA ENCUESTA)

P43. ENCUESTADOR
